# Spiropyran-based supramolecular elastomers with tuneable mechanical properties and switchable dielectric permittivity†

**DOI:** 10.1039/d4py00964a

**Published:** 2024-11-27

**Authors:** Malte Sebastian Beccard, Frank A. Nüesch, Thulasinath Raman Venkatesan, Dorina M. Opris

**Affiliations:** a Functional Polymers, Empa, Swiss Federal Laboratories for Materials Science and Technology (EMPA) 8600 Dübendorf Switzerland dorina.opris@empa.ch Thulasinath.RamanVenkatesan@empa.ch; b Eidgenössische Technische Hochschule Zürich (ETHZ) 8092 Zurich Switzerland; c Institute of Chemical Sciences and Engineering, Ecole Polytechnique Federale de Lausanne, EPFL Station 6 CH-1015 Lausanne Switzerland

## Abstract

Silicone elastomers are widely used in various applications, each demanding different properties and functionalities. To be used in such a broad spectrum, silicones with easily tunable or switchable properties are needed. We showed this is achievable with novel metallo-supramolecular polysiloxanes. Poly(dimethylsiloxane-*co*-3-aminopropylmethylsiloxane) was reacted with an epoxy-modified spiropyran (SP) in the presence of ZnCl_2_ as a catalyst. We have found that the ZnCl_2_ allows the formation of metallo-supramolecular polymers with tuneable mechanical propertiers. The influence of the amount of ZnCl_2_ used on the thermal and mechanical properties of the synthesized materials was investigated by DSC, tensile test, and DMA. The ability of SP to act not only as a physical cross-linker, but also as a molecular switch was investigated by UV-Vis spectroscopy and dielectric permittivity measurements. It was found that depending on the amount of ZnCl_2_ used, the dielectric permittivity can either increase or decrease after exposure to UV or visible light, respectively. Additionally, the developed materials can be reprocessed similarly to thermoplastic elastomers. Furthermore, their solubility can be manipulated from insoluble in practically any solvent to highly soluble by simply adding ZnCl_2_.

## Abstract 2

Functionalization of poly(dimethylsiloxane-*co*-3-aminopropylmethylsiloxane) with an epoxy-modified spiropyran using a ZnCl_2_ catalyst yields materials with tunable solubility, mechanical and thermal properties, and switchable dielectric permittivity.

## Graphical abstract

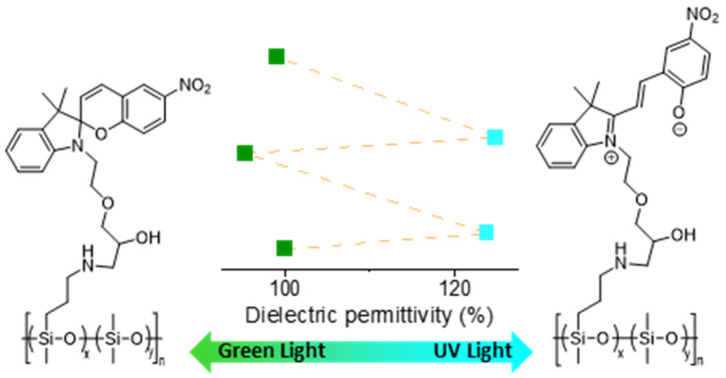

## Introduction

Silicones are inorganic polymers with a backbone consisting of alternating Si–O bonds, contributing to outstanding properties such as flexibility, low glass transition temperature (*T*_g_), good dielectric properties, chemical resistance, and physiological inertness.^1–3^ It is not surprising that silicones can be found in many applications, including bake and cookware,^4^ medicine,^5^ cosmetics, and electronics.^6^ However, as research progresses, new applications for silicones are being explored, such as active components in dielectric elastomer actuators (DEAs), sensors, generators, and stretchable electronics, to name a few. In DEAs, silicones are particularly popular due to their easy processability in thin films and low Young's modulus,^7^ as well as the possibility of attaching different side groups to the backbone, resulting in even better properties for actuation.^8^ However, to achieve the desired properties tailored for specific applications, fine-tuning must be done by different fillers,^9^ post-polymerization modification,^2^ or cross-linking density.^10^ Since most silicones have a low *T*_g_, cross-linking is needed to achieve robust materials. This is normally done by chemical cross-linking, which has the major disadvantage of leading to unprocessable and non-recyclable materials. While thermoplastic silicones are known, their synthesis is tedious, and their properties cannot be easily tuned. It would be advantageous if silicones were available and a stimulus could easily tune their properties.

To meet the requirements set, we aimed to produce a supramolecular polymer with a physical network instead of a chemical network to obtain an elastomer that can be reprocessed. Chemical networks consisting of permanent covalent cross-links afford tough and temperature-stable materials, but their permanent cross-linking has the disadvantage that it does not allow for materials to be recycled or reprocessed.^11^ A physical network, on the other hand, consists of complementary binding motifs that can associate due to non-covalent interactions but also dissociate in a given time.^12^ The resulting equilibrium can be described by the equilibrium constant:
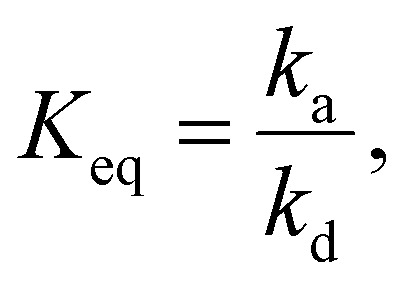
which is defined by the association rate (*k*_a_) and dissociation rate (*k*_d_) of the binding motifs. Only when a critical association rate is reached can stable supramolecular polymers be formed. The equilibrium constant depends strongly on temperature, and the equilibrium can be shifted by adding energy to the system. Consequently, the motifs dissociate faster when the supramolecular polymer is heated and it can be recycled/reprocessed.^13^ Several forces can hold a physically cross-linked polymer together. For hydrogen bonding motifs, the interactions between a hydrogen bond donor and an acceptor lead to the self-assembly of the sample.^14^ This effect can be improved by shielding the motifs with hydrophobic segments.^15^ Metal–ligand interactions can lead to supramolecular assembly, a network can be formed either by introducing several ligands into the polymer chain^16,17^ or by using a combination of metal-ion and ligand, which can form tris-complexes.^18^ Other forces include π–π-interactions, which can lead to stacking,^19,20^ or the interaction of zwitterionic motifs.^21,22^

For the design of our metallo-supramolecular polymer (MSP), spiropyran (SP) was chosen as the binding motif. SP is known for dipole–dipole interactions^23^ and for forming complexes with various metal-ions,^24^ which makes it attractive for forming MSPs. Moreover, SP is a molecular switch that reverses isomerization from a ring-closed SP form to an opened merocyanine (MC) form. Because of its switching behavior, SP is being considered for a wide range of applications, such as strain sensors,^25^ drug delivery systems,^26^ sensors for cations,^27^ and optics for photoswitchable holograms.^28,29^ The isomerization can be triggered by heat, pressure, pH change, and light.^30^ Upon irradiation with UV light, a C–O bond in the SP structure dissociates, resulting in the open zwitterionic MC form.^31^ The zwitterionic MC form has a much higher dipole moment (14–18 D) than the SP-form (4–6 D).^32^ The MC form has an extended conjugated system of π-electrons and absorbs light in the visible range. Irradiation with visible light or treatment with heat leads to further isomerization back to the SP form.^33^ SP switching ability may allow designing materials whose properties can easily be changed by an external stimulus.

Here, a poly(dimethylsiloxane-*co*-3-aminopropylmethylsiloxane) (PDMS-NH_2_) was functionalized with SP groups by reacting the primary amine with an oxirane attached to the SP. The amount of ZnCl_2_, acting as both catalyst and complexing agent, was varied and allowed us to tune the mechanical properties of the SP functionalized PDMS in a wide range. In addition, the photoswitching abilities and their impact on the dielectric properties of the synthesized materials were investigated.

## Experimental

The following reagents were used without further purification: 2,3,3-trimethyl-3*H*-indole, and 2-hydroxy-5-nitrobenzaldehyde from Apollo Scientific; 2-bromoethanol from Fisher Scientific; 2-(bromomethyl)oxirane, acetonitrile and K_2_CO_3_ from Sigma Aldrich; ZnCl_2_ from Alfa Aesar; KOH from Fluka; and tetrahydrofuran, ethanol, ethylacetate, *n*-pentane from VWR. PDMS-NH_2_ (6–7 mol% amino groups), trimethylsilyl terminated, *M*_n_ = 7200 g mol^−1^, 80–120 cst. from ABCR. The content of aminopropyl in PDMS-NH_2_ was calculated from ^1^H NMR spectrum and found to be only 5%.

^1^H and ^13^C NMR spectra were recorded on a Bruker Avance 400 NMR spectrometer at 298 K using a 5 mm broadband probe at 400.18 and 100.63 Hz. Chemical shifts are given relative to the solvents (CHCl_3_: *δ* = 7.26 ppm and 77.16 ppm; DMSO: *δ* = 2.50 ppm and 39.52 ppm). IR spectra were recorded on a Brucker Tensor 27 FT-IR spectrometer in a range of 4000–600 cm^−1^.

Thermogravimetric analysis was performed on a PerkinElmer TGA8000, heating the samples from 30 to 600 °C at 20 °C min^−1^ under air.

Differential scanning calorimetry was performed on a PerkinElmer DSC8000 in a temperature range of −80 to 80 °C with a heating/cooling rate of 20 °C min^−1^ under N_2_.

Tensile tests of dog-bone-shaped samples with a width of 2 mm and a length of 18 mm were performed on a Zwick Z010 machine. The applied preload force was 0.005 N, and the sample was stretched at a speed of 50 mm min^−1^. At least five samples were measured and averaged. Young's modulus was determined by a linear fit to the data points from 0 to 10% strain.

Dynamic mechanical analysis was performed on a RSA 3 from TA Instruments. Stripes with a width of 10 mm and a length of 12 mm were measured. The sample was measured at least three times at room temperature for each method to determine average values. For frequency-dependent measurements, a strain of 2% and a preload force of 2 g were applied, and the frequency increased from 0.05 to 10 Hz. A preload force of 2 g and a frequency of 0.1 Hz were used for the strain-dependent measurements. The measurements occurred at a strain rate of 50 mm min^−1^ from 0.05 to 10%. For the temperature-dependent measurements, a frequency of 0.1 Hz and a strain of 0.5% were used. The measurements were conducted in a temperature range from −120 to 110 °C.

A Novocontrol Alpha-A frequency analyzer was used to supply a voltage of 1 V for the dielectric relaxation spectroscopy measurements carried out between 1 and 10^6^ Hz. A Novocontrol Quatro cryosystem was used to control the sample temperature with a 2.5 K temperature step under a dry nitrogen atmosphere. For obtaining the derivative curves and fitting the dielectric data DCALC program developed by Wübbenhorst was used.^34,35^

UV-Vis measurements were conducted with a Varian Cary 50 UV-Vis spectrophotometer ranging from 300 to 800 nm. As a UV source for the irradiation experiments, 6 LEDs from Distrelec with a wavelength of 365 nm and a radiant power of 1 W per LED were mounted on a PCB plate on a surface of 2 cm^2^. For the green light, 6 LEDs from Distrelec, with a wavelength of 505 nm and a radiant power of 1.1 W, were mounted on a PCB plate on a surface of 2 cm^2^.

The synthesis of compounds 1, 2, SP-OH, and SP-Ep was carried out following established literature^36,37^ and a comprehensive description is available in the ESI section.†

### Synthesis of PDMS-SP-Ep-(Zn)

3′,3′-Dimethyl-6-nitro-1′-(2-(oxiran-2-ylmethoxy)ethyl)spiro[chromene-2,2′-indoline] (0.26 g, 0.63 mmol), varying amounts of ZnCl_2_, and PDMS-NH_2_ (1 mL) were dissolved in THF (20 mL) to give PDMS-SP-Ep-(Zn) with different molar ratios of SP to ZnCl_2_ (*x* : *y*). After refluxing the solution overnight, the solvent was evaporated, and the polymer was dried in the vacuum oven at 65 °C. The product was used without further purification steps and was obtained as an orange-brownish solid. Sample 1 : 0.01 was refluxed for 2 days. Sample 1 : 1.12 was obtained by mixing a 1 : 1 ratio of two solutions of samples 1 : 1 and 1 : 1.25. The complete functionalization was confirmed by the absence of the oxirane band at 908 cm^−1^ (Fig. S1†). ^1^H NMR spectroscopy was conducted on the soluble sample 1 : 1 (Fig. S2†), but the resolution was poor. Nevertheless, the signals from the SP-Ep showed peak broadening, and the NH_2_ peak at 2.2 ppm disappeared, indicating complete functionalization.

### Preparation of the thin films

PDMS-SP-Ep-(Zn) was placed between two Teflon films attached to metal plates and separated by spacers with a thickness of 40 μm or 200 μm, respectively. The films were pressed with 3 t at 100 °C for three minutes. For 1 : 0.01, the samples were pressed for 2 hours.

### UV-Vis spectroscopy

The samples 1 : 1; 1 : 0.5 and 1 : 0.01 were pressed into a film, but without spacers. After each measurement, the samples were exposed to the light sources at a distance of 1 cm and in steps of 2 min for green light and 10 s for UV light.

### Switch in permittivity

The samples 1 : 1, 1 : 0.5, and 1 : 0.01 were pressed into films with thickness >40 μm. Dielectric permittivity measurements were conducted three times for each sample. Exposure to UV light lasted 2 min, while treatment with green light took 10 min. The samples were placed at a distance of 1 cm from the light source.

## Results and discussion

**Scheme 1:**
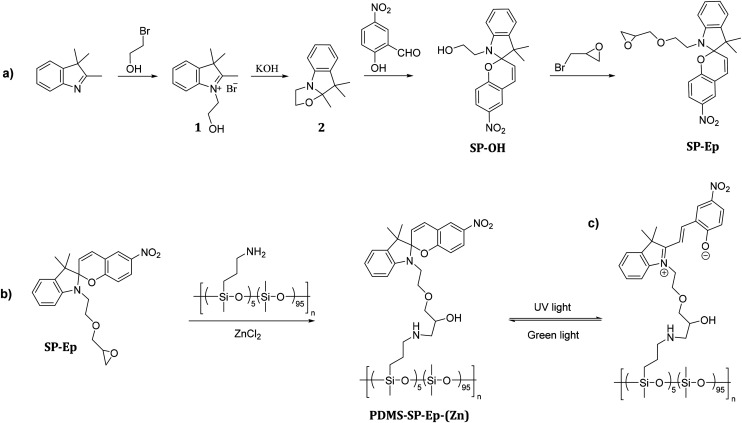
Synthetic pathway for the photoswitch SP-Ep (a) and post-polymerization modification of PDMS-NH_2_ with SP-Ep catalyzed by ZnCl_2_ (b), and conversion of SP to MC (c).

Scheme 1a outlines the synthetic strategy employed for the preparation of SP-Ep. In the first step, 2,3,3-trimethyl-3*H*-indole was reacted with 2-bromoethanol, yielding product 1. Treatment of 1 with KOH afforded product 2, which was subsequently reacted with 2-hydroxy-5-nitrobenzaldehyde to afford SP-OH. Finally, SP-Ep was synthesized by reacting SP-OH with 2-(bromomethyl)oxirane. The composition of commercial PDMS-NH_2_ with 5 mol% of amino functional groups was determined by ^1^H NMR spectroscopy. Based on this information, the amount of SP-Ep required for complete functionalization of the polymer was calculated using the molecular weight of a theoretical average repeating unit (RU):[0.95 × *M*(RU_PDMS_) + 0.05 × *M*(RU_NH_2_ functionalized PDMS_)].

Functionalization of PDMS-NH_2_ with the chromophore SP-Ep was achieved using ZnCl_2_ as a catalyst (Scheme 1b). Immediately after the synthesis, PDMS-SP-Ep was well soluble in THF and chloroform. However, after washing it with water, and thus ZnCl_2_ removal, the polymer turned insoluble. The addition of a small amount of ZnCl_2_ rendered the polymer soluble again. Indeed, ZnCl_2_ was crucial for both the reaction between PDMS-NH_2_ and SP-Ep and for forming solid supramolecular polymers (SMPs). While the unreacted PDMS-NH_2_ and SP-Ep mixture 1 : 0 and the PDMS-NH_2_ and ZnCl_2_ mixture remained liquid, PDMS-SP-Ep and ZnCl_2_ mixture solidified. This suggests that functionalization with the chromophore leads to non-covalent interactions responsible for the SMPs’ solidification. Therefore, we synthesized materials with varying SP-EP to ZnCl_2_ ratios of 1 : 2, 1 : 1.12, 1 : 1, 1 : 0.5, 1 : 0.1, and 1 : 0.01, named the resulting materials PDMS-SP-Ep (*x* : *y*), and investigated the impact of ZnCl_2_ content on the solubility and appearance of different materials (Table 1). Thus, all prepared materials have the same polysiloxane backbone and content of SP-Ep, but differ in their relative molar ratio of SP-Ep to ZnCl_2_. This ratio significantly impacts the solubility of the SMPs in THF and CHCl_3_. Specifically, a SP-Ep to ZnCl_2_ ratio of 1 : 05 or even higher amounts of ZnCl_2_ result in soluble SMPs, while a decrease of ZnCl_2_ leads to insoluble SMPs. Experiments showed that the material 1 : 0.01, initially insoluble in THF and CHCl_3_, could be dissolved by adding small amounts of ZnCl_2_ to the solution (Fig. S3†). However, an excess of ZnCl_2_, as in sample 1 : 2, leads to a viscous liquid.

**Table 1:** SMPs with different SP-Ep : ZnCl_2_ (*x* : *y*) ratios, composition, solubility in THF or chloroform, and appearance

Sample	Eq. SP-Ep per eq. NH_2_-group	Eq. ZnCl_2_ per eq. NH_2_-group	Soluble in THF/CHCl_3_	Appearance
1 : 1.25	1	1.25	Yes	Highly viscous
1 : 1.12	1	1.12	Yes	Solid
1 : 1	1	1	Yes	Solid
1 : 0.5	1	0.5	Yes	Solid
1 : 0.1	1	0.1	No	Solid
1 : 0.01	1	0.01	No	Solid

**Fig. 1:**
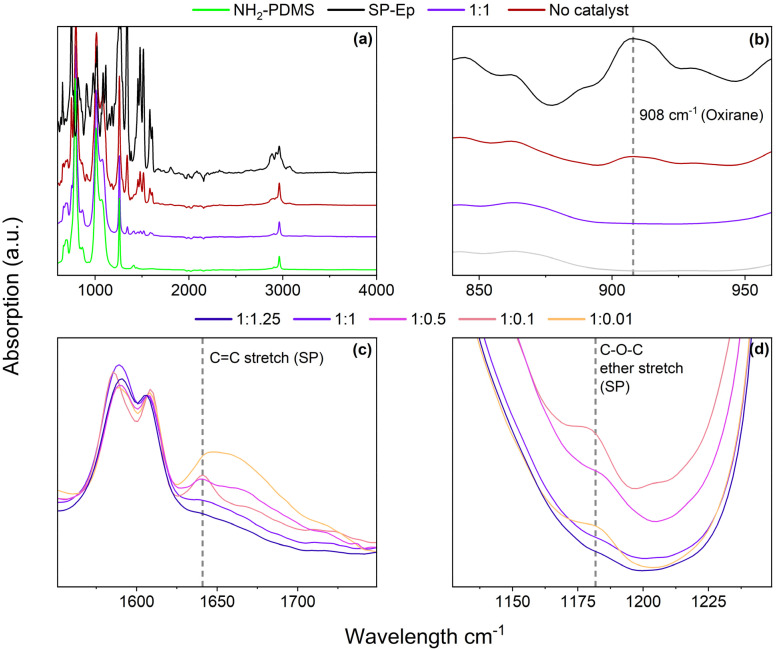
(a) IR spectra of PDMS-NH_2_, SP-Ep, and SMP 1 : 1 with equimolar amount of SP-Ep to ZnCl_2_ and sample 1 : 0 without ZnCl_2_. (b) the functionalization of PDMS-NH_2_ with SP-Ep is confirmed by the disappearance of the peak at 908 cm^−1^ characteristic of NH_2_ groups. (c) and (d) Effect of ZnCl_2_ on the isomeric state of SP-Ep is demonstrated by the differences in the IR spectra of the SP and MC forms.

To confirm that SP-Ep was indeed chemically bonded to the polysiloxane chain, both ^1^H NMR spectroscopy and IR spectroscopy were used. ^1^H NMR spectroscopy was used for the samples soluble in CHCl_3_. Complex spectra were observed due to comparable low amounts of SP-Ep and complex formation with the Zn^2+^ ions. For the sample 1 : 1, it was observed that the NH_2_ peak at 2.2 ppm disappeared (Fig. S2†), while the signals for the propyl chain remained at the same chemical shift, indicating successful attachment of SP-Ep to the main chain. IR spectroscopy (Fig. 1a) allowed the complete reaction of SP-Ep to be monitored by analyzing the absorbance peak of the oxirane at 908 cm^−1^ (Fig. 1b and S9†). The peak disappeared in the samples where ZnCl_2_ was used as a catalyst, while the peak persisted in the mixture of PDMS-NH_2_ and SP-Ep without the catalyst. The IR absorption bands from SP-Ep provide a better understanding of the influence of ZnCl_2_ on the SMPs. Fries *et al.* found significant changes in the IR spectra between the closed SP and open MC forms. For instance, the stretching band of C–N or O–C–N disappears after opening SP up to the MC form, while other bands like the stretch of C–O^−^ and C=N^+^ are only present in the MC form.^38^ Sample 1 : 0.01 exhibited a band at 1641 cm^−1^. The band was weaker in samples 1 : 0.1 and 1 : 0.5 (Fig. 1c). As the amount of ZnCl_2_ in the sample increased, the band weakened and ultimately disappeared entirely in sample 1 : 1.25. The band was assigned to the C=C stretch of the SP. Samples 1 : 0.01 and 1 : 0.1, with low amounts of ZnCl_2_, exhibit an absorption band at 1181 cm^−1^ (Fig. 1d). This band becomes less pronounced in sample 1 : 0.5 and disappears in samples 1 : 1 and 1 : 1.25 with higher amounts of ZnCl_2_. The observed band corresponds to the asymmetrical C–O–C ether stretch, which is present only in the SP-isomer and not in the MC-isomer. The results suggest that the presence of ZnCl_2_ induces SP to open up, presumably to form complexes. In the absence of ZnCl_2_, the closed SP form is favored.

**Fig. 2:**
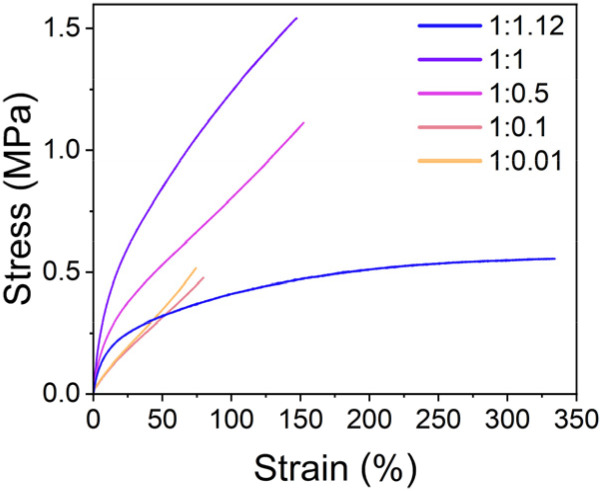
Representative curves for the tensile test of samples with different SP-Ep : ZnCl_2_ (*x* : *y*) ratios.

Tensile tests were performed to investigate the mechanical properties of samples with different SP-Ep to ZnCl_2_ ratios. Fig. 2 and Table 2 illustrate the relationship between the mechanical properties and the ratios of ZnCl_2_ to SP-Ep for different samples. Samples of SMPs 1 : 0.01 and 1 : 0.1 with a low content of ZnCl_2_ exhibit a low strain at break, low elastic modulus, and low tensile strength. Despite the low salt content and the absence of any chemical cross-links, the materials are elastic. These results suggest that SP-Ep functions as a physical cross-linker. As the amount of ZnCl_2_ was increased, an improvement in mechanical properties was observed. Increasing the amount of ZnCl_2_ in the materials results in an increase in stress at break from 0.52 to 0.45, 1.09, and 1.55 MPa, an increase in Young's modulus from 0.87, 0.84, 2.51, and 3.36 MPa and an increase in toughness from 0.20, 0.17, 0.98 to 1.44 MPa for SMPs 1 : 0.01, 1 : 0.10, 1 : 0.5 and 1 : 1, respectively. These results indicate that the mechanical properties of the SMPs are weakened for samples with reduced amounts of complexes acting as physical cross-links. The average elongation at break remains constant at 142 and 148% for samples 1 : 1 and 1 : 0.5. However, a further reduction in ZnCl_2_ for the 1 : 0.1 and 1 : 0.01 samples reduces elongation at break to 70 and 75%, respectively. These results suggest that a certain concentration of ZnCl_2_ is required for a good network of physical cross-links (1 : 0.5) leading to good mechanical properties. However, after a critical concentration of 1 : 1, a further increase in ZnCl_2_ leads to a drastic increase in elongation and a decrease in Young's modulus. Even a small excess of ZnCl_2_ (1 : 1.12) results in more than twice the strain at break (337%) and a large reduction in the stress at break (0.56 MPa) and Young's modulus (1.64 MPa). Interestingly, the toughness remains the same (1.47 MPa) when compared to the 1 : 1 sample. Adding ZnCl_2_ has a plasticizing effect, allowing deformation at lower stresses.

**Table 2:** Data for strain at break, stress at break Young's modulus, and toughness of samples with different SP-Ep : ZnCl_2_ (*x* : *y*) ratios

Sample	*ε*_(Break)_ a (%)	*δ*_(Break)_ a (MPa)	*Y*_10%_ a ^,^ b (MPa)	Toughness^a^ (MPa)
1 : 1.12	337 ± 32	0.56 ± 0.06	1.64 ± 0.11	1.47 ± 0.24
1 : 1	142 ± 8	1.55 ± 0.06	3.36 ± 0.15	1.44 ± 0.15
1 : 0.5	148 ± 9	1.09 ± 0.06	2.51 ± 0.10	0.98 ± 0.09
1 : 0.1	70 ± 10	0.45 ± 0.05	0.84 ± 0.05	0.17 ± 0.04
1 : 0.01	75 ± 3	0.52 ± 0.03	0.87 ± 0.02	0.20 ± 0.01

a At least six values were taken.

b Calculated by linear fitting the data points from 0 to 10% strain.

**Fig. 3:**
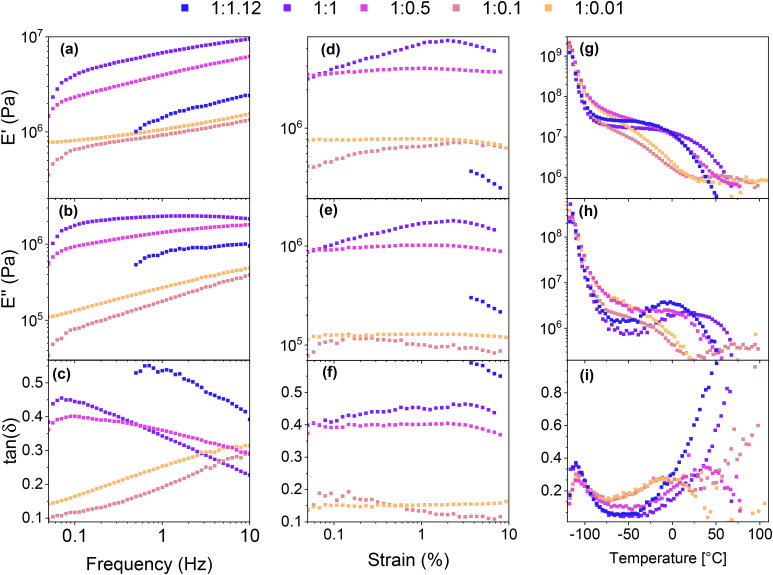
Dynamic mechanical analysis of different samples: the storage modulus, loss modulus, and tan(*δ*) in the frequency mode (a), (b), and (c); strain sweep (d), (e), and (f); and in dependence of the temperature (g), (h), and (i). All curves represent the average of at least three measurements.

Frequency-dependent viscoelastic measurements were carried out at room temperature to investigate the relationship between the mechanical properties and the SP-Ep : ZnCl_2_ ratio. Sample 1 : 1.12 could not be measured at low frequencies due to unstable values. All samples exhibited a storage modulus *E*′ higher than the loss modulus *E*′′ over the frequency range investigated. With a decreasing amount of ZnCl_2_, *E*′ and *E*′′ decreased, showing a similar behavior to the tensile test. For the polymer with a high amount of ZnCl_2_ (1 : 1.12, 1 : 1, and 1 : 0.5), tan(*δ*) decreased with increasing frequency (Fig. 3a–c), indicating a better elastomeric behavior at high frequencies. The opposite trend was observed in the samples with lower amounts of ZnCl_2_ (1 : 0.1, 1 : 0.01), indicating that the viscous response becomes more pronounced at higher frequencies. These observations suggest that at room temperature, relatively low amounts of the complex are sufficient to yield solid-like behavior at low frequencies. However, this response becomes less pronounced as the frequency increases due to the inability of the physical cross-links between the SPs to store and release energy rapidly. On the other hand, as the ZnCl_2_ content increases, more complexes are formed and the materials can store more energy at higher frequencies. This is evidenced by the decreased tan(*δ*) for the 1 : 1.12, 1 : 1, and 1 : 0.5 samples. One potential explanation for this behavior is that at low ZnCl_2_ amounts, intermolecular complexes between two distinct chains are the predominant species, thereby leading to higher losses at higher frequencies, whereas, at higher concentrations, intramolecular complexes within the same chain are formed, which lowers the elastic modulus at increased frequencies.

Strain-dependent DMA measurements were performed to investigate the elastic properties of the different samples (Fig. 3d–f). Sample 1 : 1.12 could only be measured at higher strains as the losses were too high for the instrument to measure at lower strains. The results show that similar to the frequency-dependent measurements, the storage modulus increased with increasing ZnCl_2_ content (from 1 : 0.01 and 1 : 0.1 to 1 : 1). However, for the 1 : 1.12 sample with a small excess of ZnCl_2_ to SP, the modulus decreased. Interestingly, for all samples tested, the influence of the strain on *E*′ and *E*′′ was limited. Notably, for samples with low amounts of ZnCl_2_, both the modulus and tan(*δ*) exhibited a notable reduction compared to samples with a higher amount of the complex. This finding is consistent with the results of the frequency sweep, which demonstrated that samples with low amounts of complex exhibited a lower tan(*δ*) at low frequencies. The results provide further evidence supporting the hypothesis that two distinct types of complex cross-links are present. In samples with a low complex concentration (1 : 0.01 and 1 : 0.1), intermolecular cross-links are formed, resulting in low losses and a lower modulus. Conversely, higher complex amounts lead to intramolecular cross-links, which exhibit a higher modulus and losses due to their susceptibility to rupture under high strain.

The thermal stability of the samples was investigated using TGA (Fig. S4a†). All samples exhibit great stability up to 150 °C and show a mass loss of 2% above 170 °C.

Differential scanning calorimetry (DSC) measurements were performed to investigate the thermal transitions of SP-Ep and PDMS-SP-Ep. Upon cooling, pure SP-Ep shows a broad exothermal peak at 36 °C, attributed to a crystallization process (Fig. S4b†). Upon heating, an endothermal peak is observed at 45 °C, corresponding to the melting of the crystalline phase (Fig. S4c†). In fact, no thermal transitions were detected between −80 and 80 °C for any of the samples, neither on cooling nor on heating, suggesting that the grafting of SP-Ep onto the PDMS chain prevents SP-Ep crystallization (Fig. S4d and e†). Even sample 1 : 0.01, with the lowest amount of ZnCl_2_, does not crystallize. All materials exhibited an elastic behavior at room temperature, suggesting that all should have a *T*_g_ below −80 °C and another transition temperature above 80 °C, responsible for the physical cross-links. Unfortunately, our DSC setup did not allow measurements to be conducted at even lower temperatures. Therefore, to investigate the temperature-related transitions in the polymer structure, temperature-dependent DMA measurements were conducted for samples 1 : 1.12, 1 : 1, 1 : 0.5, 1 : 0.1, and 1 : 0.01 (Fig. 3g–i). All samples exhibited a decrease in modulus as temperatures increased from −115 °C. The maximum in tan(*δ*) was observed at temperatures around −110 °C (for 1 : 1.12 at −111 °C, 1 : 1 at −110 °C, 1 : 0.5 at −110 °C, 1 : 0.1 at −109 °C, and 1 : 0.01 at −110 °C) (Table 3). This maximum in tan(*δ*) corresponds to the *T*_g_ of the PDMS phase and is independent of the amount of ZnCl_2_. As the temperature increased, *E*′ decreased for all samples. A plateau in *E*′ was reached for samples with a high amount of ZnCl_2_, however, samples 1 : 0.1 and 1 : 0.01 exhibited a steady decrease in *E*′ up to about 25 °C where *E*′ reached a plateau. This suggests that a complex is responsible for the plateau in the low-temperature range. DMA measurements suggest the presence of a second *T*_g_, which was strongly dependent on the content of ZnCl_2_ in the SMPs. With an increasing amount of ZnCl_2_, the peak of tan(*δ*) is shifted to higher temperatures, from around −10 °C for 1 : 0.01 and 1 : 0.1 to about 37 °C for 1 : 0.5, and goes up to higher temperatures for the 1 : 1. Thus, tan(*δ*) increased strongly for samples with high amounts of ZnCl_2_ to elevated temperatures. For sample 1 : 1.12, tan(*δ*) reached a value of 1 at temperatures of 44 °C. Higher amounts of the complex led to a higher storage modulus at 25 °C, while an excess of ZnCl_2_ resulted in a lower modulus. These findings are consistent with the results from tensile testing and frequency sweep-dependent DMA measurements. Thus, at room temperatures, sample 1 : 1 showed the highest storage modulus of 7.3 MPa, followed by 1 : 0.5 with 3.71 MPa, 1 : 1.12 with 2.97 MPa, 1 : 0.1 with 0.95 MPa, and 1 : 0.01 with 0.92 MPa, respectively (Table 3). The aforementioned observations also explain why the polymers exhibit elastomeric properties but can be processed like a thermoplastic polymer at elevated temperatures. The relatively low tan(*δ*) at room temperature provides stability and an elastomeric behavior, but as temperature increases, the material softens and can be melt pressed, similarly to a thermoplastic elastomer.

**Table 3:** Glass transition temperature (*T*_g_) and results from temperature and strain-dependent DMA measurements, including storage and loss modulus at different temperatures/frequencies

Sample	*T*_g_ (°C)	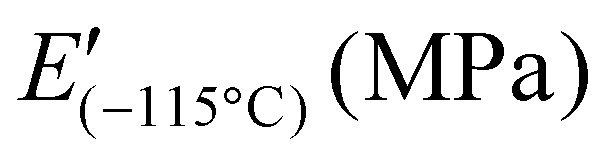	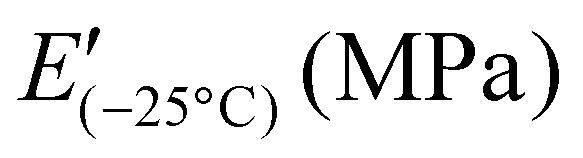	*T*_(tan(*δ*)=1)_ (MPa)	*E*′; *E*′′ at 0.1 Hz (MPa)	*E*′; *E*′′ at 1 Hz (MPa)	*E*′; *E*′′ at 10 Hz (MPa)
1 : 1.12	−111 ± 1	1360 ± 70	2.97 ± 0.23	44 ± 6	Not determined	1.4 ± 0.2; *0.76* ± *0.14*	2.4 ± 0.2; *0.95* ± *0.06*
1 : 1	−110 ± 1	1190 ± 70	7.3 ± 0.8	66 ± 6	4.0 ± 0.2; *1.8* ± *0.1*	6.8 ± 0.3; *2.3* ± *0.1*	9.5 ± 0.4; *2.2* ± *0.1*
1 : 0.5	−110 ± 1	1690 ± 170	3.71 ± 0.08	Above 75^a^	2.3 ± 0.1; *0.93* ± *0.03*	4.0 ± 0.1; *1.4* ± *0.1*	6.2 ± 0.3; *1.8* ± *0.1*
1 : 0.1	−109 ± 1	1300 ± 110	0.95 ± 0.05	Above 97^a^	0.66 ± 0.04; *0.077* ± *0.001*	0.93 ± 0.03; *0.18* ± *0.01*	1.35 ± 0.06; *0.40* ± *0.02*
1 : 0.01	−110 ± 1	1590 ± 110	0.92 ± 0.06	Not determined^b^	0.82 ± 0.02; *0.13* ± *0.01*	1.08 ± 0.03; *0.28* ± *0.01*	1.56 ± 0.05; *0.49* ± *0.02*

a Not for every sample tan(*δ*) = 1 could be determined until *T* = 110 °C.

b No sample showed a tan(*δ*) = 1 up to *T* = 110 °C.

**Fig. 4:**
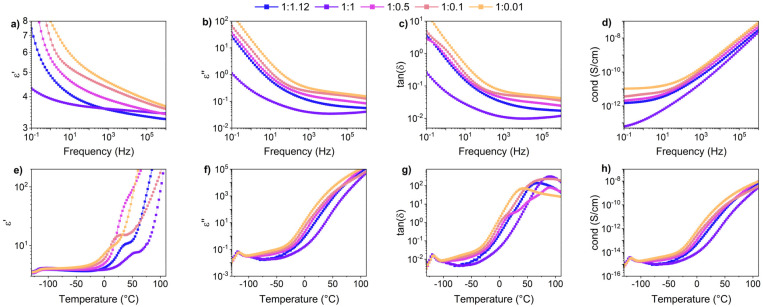
Dielectric spectroscopy measurements of samples with different amounts of ZnCl_2_ and pure ligand. Given are the frequency-dependent changes in dielectric permittivity (*ε*′) (a), dielectric loss (*ε*′′) (b), dissipation factor (tan *δ*) (c), and conductivity (d). (e)–(h) show the temperature-dependent changes in *ε*′ (e), *ε*′′ (f), tan *δ* (g), and conductivity (h) at 0.1 Hz.

Dielectric properties were measured for samples with different amounts of ZnCl_2_. As shown in Fig. 4a–c, for all samples, permittivity (*ε*′) and dielectric losses (*ε*′′) increased with decreasing frequency, while in contrast, conductivity (Fig. 4d) increased with increasing frequency. Fig. 4a shows that at 1 kHz the dielectric permittivity of all materials is higher than pure PDMS (*ε*′ = 3), even if the polysiloxane backbone was modified with only 5 mol% SP-Ep side chains.^39^ The permittivity at 1 kHz decreases with an increasing amount of ZnCl_2_ in the sample from 4.7 for 1 : 0.01, to 4.4 for 1 : 0.1, to 3.9 for 1 : 0.5, to 3.6 for 1 : 1 and 1 : 1.12. Similarly, a decrease in *ε*′′ and conductivity is observed with a decreasing amount of ZnCl_2_. It can be concluded that the dipoles of SP-Ep interact with the Zn-ion and the formed SP-Ep-Zn complex is less susceptible to polarization.

Dielectric spectroscopy measurements as a function of temperature can help study the transitions occurring in the sample and complement the DMA measurements. Fig. 4e–h lists the real and imaginary components of the complex dielectric permittivity, including the dissipation factor tan *δ* and the conductivity of all the samples with varying amounts of ZnCl_2_, measured from −130 °C to 110 °C at a constant frequency of 0.1 Hz. From the *ε*′ plot, all samples show a step increase in permittivity below −100 °C. This is manifested as a peak in both dielectric loss and tan *δ*. Comparing with the data from DMA, this should correspond to the glass-transition relaxation process of PDMS and, as expected, is independent of the amount of ZnCl_2_. Above the first *T*_g_ loss peaks, we see an additional shoulder for high concentration of ZnCl_2_ (1 : 1 and 1 : 1.12). The shoulder becomes broader for lower concentrations of ZnCl_2_.

From DMA measurements, we expect to see a relaxation around −25 °C for the samples with low ZnCl_2_ content, moving progressively to higher temperatures with increasing ZnCl_2_. For 1 : 0.01 sample, we observed a relaxation process reflected by the shoulder in the permittivity spectrum at 10 °C. This relaxation process is more clearly seen in the 1 : 0.1 sample at 25 °C. A further increase in the ZnCl_2_ concentration shifts the relaxation to higher temperatures, albeit a lower change in permittivity during this process. While, this process is clearly visible in permittivity, it cannot be easily identified in their corresponding loss plots. This is due to the enhanced conductivity of all samples above −25 °C. Further analysis is therefore required to explain the various transitions observed in dielectric spectroscopy.

Sample 1 : 0.1 was chosen for detailed analysis. As a first step, contributions from d.c. conductivity (ohmic conduction) was removed by performing a frequency derivative of permittivity, as stated in eqn (1).1
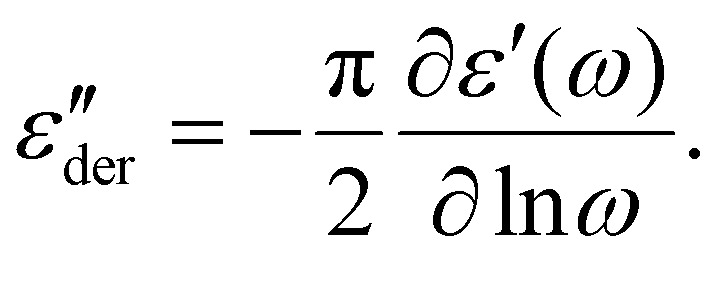


**Fig. 5:**
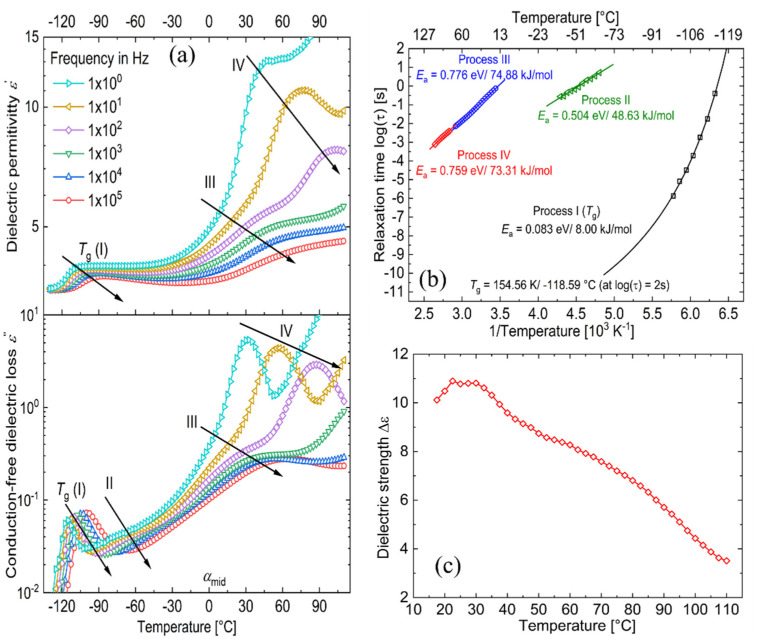
Dielectric analysis of a 1 : 0.1 SP-Ep : ZnCl_2_ sample (a) real part of dielectric permittivity (*ε*′) and d.c. conduction-free dielectric loss (
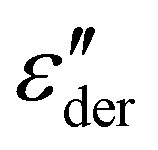
) as a function of temperature at selected frequencies. (b) Arrhenius relaxation plot of all the observed relaxations. (c) Dielectric strength (Δ*ε*) of the observed relaxations between 20 and 110 °C.

**Fig. 6:**
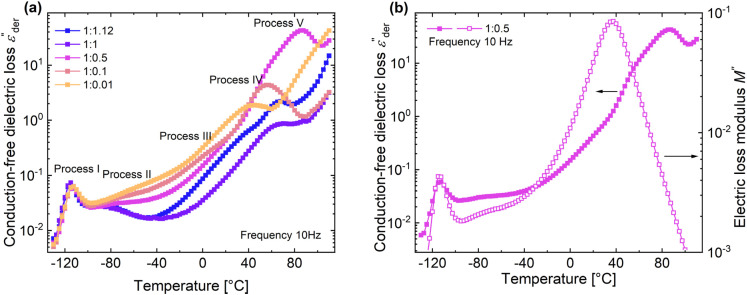
(a) D.C. conduction-free dielectric loss (
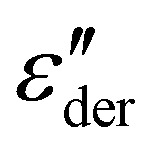
) curves of different SP-Ep : ZnCl_2_ samples as a function of temperature at 10 Hz. (b) 
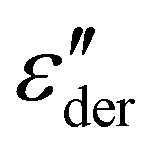
 and dielectric loss modulus *M*′′ temperature of 1 : 0.5 and 1 : 0.1 samples as a function of temperature at 10 Hz.

Fig. 5a shows the d.c. conduction-free dielectric loss (
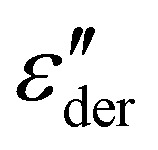
) and permittivity (*ε*′) plotted as a function of temperature at selected frequencies. Comparing 
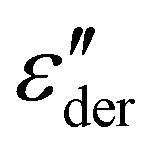
 curves to Fig. 4f, in addition to the two transitions (process I and II) found at lower temperatures, we observe two additional processes above 0 °C named as process III and IV. As stated earlier, process I is assigned to a glass-transition process. The peaks shift to higher temperatures with increased frequency, as expected in a relaxation process.^40^ The Havriliak–Negami (HN) function^41^ was used to fit the loss peaks of the process I. The relaxation times obtained from peak fitting are plotted in Fig. 5b. The fit is non-linear and obeys Vogel–Fulcher–Tammann (VFT) law affirming a glass-transition relaxation.^41^ The dynamic *T*_g_ can be calculated by extrapolating the curve to a relaxation time of 100 s (log *τ* = 2 s),^42^ which yields a temperature of −118.6 °C, agreeing well with the value obtained from DMA. Similar to process I, the other processes were also subjected to a HN-fit, and the corresponding relaxation map is plotted in Fig. 5b. In contrast to process I, all the other processes result in a linear fit obeying an Arrhenius-type equation.^41^ Since all these processes also shift with frequency and the polymer samples are amorphous, structural transition processes such as melting and crystalline phase change can be excluded.^40^ To obtain an overview of these different processes across the samples with different amounts of ZnCl_2_, 
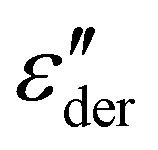
 curves of all the samples as a function of temperature at a fixed frequency of 10 Hz are shown in Fig. 6a. Looking into process II, we find that the relaxation is stronger for samples with a higher amount of ZnCl_2_ and progressively becomes weaker with decreasing amounts of ZnCl_2_. Correlating this with the change in permittivity during this temperature range for the different samples (Fig. 4e), we find that samples with higher amount of ZnCl_2_, *i.e.*, higher amount of complex formation (1 : 1.12 and 1 : 1), show a dip in permittivity, while the SP-Ep with lower fraction of ZnCl_2_ (1 : 0.1 and 1 : 0.01) show a small increase. As previously stated, the complexation of SP-Ep by ZnCl_2_ reduces the permittivity of the samples, and hence, process II can be assigned to the relaxation of these complex units.

Moving on to process III, looking at the loss curves in Fig. 5a, it appears as a shoulder at a frequency of 10 Hz and transforms into a peak at higher frequencies. From Fig. 6a, we also notice that the process is stronger and occurs at a higher temperature for samples with higher ZnCl_2_. This relaxation can be directly correlated with the loss modulus peaks occurring between −25 °C and +50 °C across different samples in DMA measurements (Fig. 3h). As mentioned earlier, the mechanical failure of samples above this transition hints at the disassociation of the complex in the samples. This also explains the higher temperature at which the shoulder is observed for samples with higher ZnCl_2_. A similar trend is observed with the process IV peaks, and by looking at the Arrhenius plots for both process III and IV in Fig. 5b, we observe similar activation energies and distribution of relaxation times. All these suggest that both these processes are related and can be assigned to the dissociation of the two types of complexes hypothesized to exist in the samples. Plotting the dielectric strengths (Δ*ε*) of both these processes (Fig. 5c) obtained from the HN fitting of a 1 : 0.1 sample reveals a continuous curve with a peak at 27.5 °C and a shoulder at 77.5 °C corresponding to processes III and IV, respectively. Process III can be assigned to the intermolecular complexes, which are thermally less stable than intramolecular complexes, which dissociate around 80 °C (process IV).

For a 1 : 0.5 sample, above process IV, we can observe yet another peak at 87.5 °C in Fig. 6a. Since, the complexes already dissociate at this temperature, the ions can move to their respective counter electrodes, resulting in electrode polarization.^43^ To check this, the imaginary part of complex electric modulus *M*′′ = *ε*′′/*ε*′^2^ + *ε*′′^2^ is plotted in Fig. 6b along with 
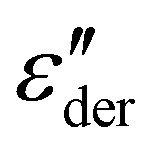
 for a 1 : 0.5 sample. The advantage of using a *M*′′ loss plot is suppressing electrode polarization.^44,45^ From the figure, we clearly observe the absence of process V in the *M*′′ loss curve, confirming it to be electrode polarization. Such a process can be expected to be observed at higher temperatures for the rest of the samples, indicated by the increase in losses in Fig. 6a. On the other hand, the peak observed at 37.5 °C in the *M*′′ loss curve can be delegated to the shoulder of process IV relaxation observed in 
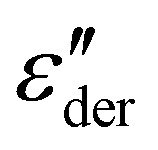
.

Using dielectric spectroscopy and detailed analysis, it is possible to identify the different relaxations taking place in the samples under investigation. On heating above −110 °C, we first observe the glass transition at low temperatures, immediately followed by the relaxation of complexes. Above −30 °C, we observe the disassociation of complexes at two different temperatures. While process III is observed as a shoulder, process IV is observed as a peak. On further heating the samples to higher temperatures above 80 °C, the disassociated ions lead to the observation of electrode polarization. Except for glass transition, all other processes depend on the concentration of ZnCl_2_ in the sample. In general, a higher fraction of ZnCl_2_ increases the strength of these processes. While the relaxation of the complexes (process II) in samples with a higher presence of ZnCl_2_ is observed at a lower temperature, the opposite holds for processes III and IV.

**Fig. 7:**
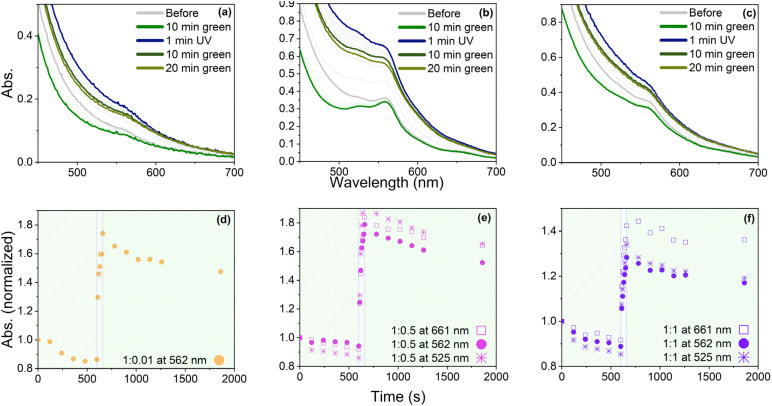
Absorption of sample 1 : 0.01 (a), 1 : 0.5 (b), and 1 : 1 (c) before irradiation, after 10 min irradiation with green light, 1 min irradiation with UV light, 10 min green light, and after a total of 20 min green light. (d, e and f) Absorption at selected absorption bands against the irradiation time with green and UV light before irradiation, after 10 min irradiation with green light (in 2 min steps), 1 min irradiation with UV light (10 s steps), 10 min green light (2 min steps), and after a total of 20 min green light.

To investigate the light-triggered switching capabilities of the synthesized materials, UV-Vis spectra of three selected samples 1 : 1, 1 : 0.5, and 1 : 0.01 were recorded before and after exposure to green and UV light (Fig. 7a–c). Sample 1 : 0.01 (Fig. 7a) showed one absorption band at 562 nm, whereas samples 1 : 0.5 and 1 : 1 (Fig. 7b and c) exhibited three absorption bands, two weak ones at 661 and 525 nm, and a stronger one at 525 nm, with 1 : 0.5 showing a more pronounced band at 525 and 661 nm. These results suggest that the 661 and 525 nm bands result from complex formation. To ensure that SP-Ep did not contain open MC form, all samples were first exposed to green light at a wavelength of 505 nm for 10 min. The resulting decrease in absorption suggests that some SP-Ep had already undergone isomerization to the open MC form and subsequently switched back to the closed form upon irradiation with green light. Nevertheless, a small peak at 562 nm was observed in all samples, which was attributed to the MC form. This phenomenon was more pronounced in samples 1 : 1 and 1 : 0.5, which had a higher ZnCl_2_ content. This suggests that ZnCl_2_ stabilizes the MC form and deactivates the switch back to the SP form. The absorbance significantly increased after applying UV light with a wavelength of 365 nm for 1 min. The highest increase was observed for 1 : 0.5 (Fig. 7e), followed by 1 : 0.01 (Fig. 7d), and the smallest change in absorbance was observed for 1 : 1 (Fig. 7f). Upon exposure to green light for 20 min, the absorbance decreased, but it was impossible to re-obtain the original spectra, which would have indicated a complete switch back. The results suggest that despite being chemically bonded to the polymer and incorporated into a complex, SP-Ep still exhibits photo-switching behavior. However, photoisomerization towards the open MC form is faster and more efficient than the switch back with a green light, which was incomplete. Several possible explanations include photo-induced oxidation processes resulting from highly energetic UV light exposure. Additionally, metal ions and stacking phenomena in the physical cross-links may stabilize the opened form. It is known from the literature^23^ that the open MC form can be stacked. Eckhardt *et al.* also reported that the ability to shift back into the closed-ring form depends on the degree of MC association and sometimes cannot be changed back without degradation.^46^

**Fig. 8:**
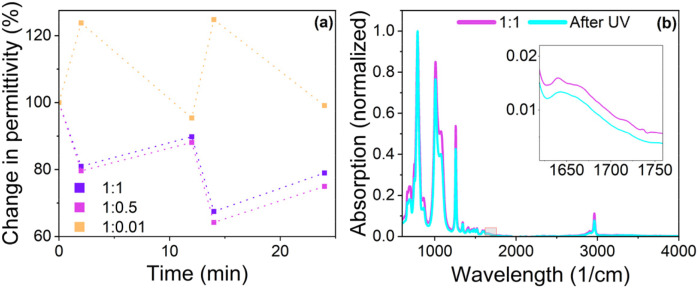
Relative change in permittivity at 1 kHz of sample 1 : 1, 1 : 0.5, and 1 : 0.01 after exposure to UV for 2 min followed by green light 10 min (2 cycles) (a). Absorption spectra of 1 : 0.5 before and after UV light (b).

To further investigate changes in the properties of the samples, permittivity was measured before and after exposure to UV and green light (Fig. 8a). The initial value before light exposure was set as 100%. After exposure for 2 min with UV light, the values decreased to 81 and 80% for 1 : 1 and 1 : 0.5, respectively. Exposure to green light for 10 min led to a permittivity value of up to 90 and 88% of the original value for 1 : 1 and 1 : 0.5 samples, respectively. Repeating the procedure resulted in a decrease to 67 and 64%, followed by an increase after exposure to UV light to 79 and 75%, respectively. Even though the switching behavior can be repeated, the original permittivity values cannot be restored. On the contrary, exposure of the 1 : 0.01 sample to UV light for 2 min resulted in an increase in permittivity of +24%, followed by a decrease to 95% after exposure to green light for 10 min. This switching behavior could be repeated, and in the second run, exposure to UV light led to an increase of up to +25%, followed by a decrease to 99%. Hence, for sample 1 : 0.01, exposure to UV light is accompanied by an increase in permittivity, while exposure to green light can restore values close to the original ones. We also observed that sample handling became more difficult for all materials after exposure to UV light, possibly due to changes in the internal structure caused by stacking phenomena, as discussed earlier. Comparing the results for 1 : 1 and 1 : 0.5 with those of 1 : 0.01, it can be seen that the presence of a complex in the sample significantly influences the photo-switching behavior. For sample 1 : 0.01, UV light-induced ring opening of the chromophore increases the dielectric permittivity. However, the dielectric permittivity for the ring-opened MC form in 1 : 0.5 and 1 : 1 decreased. The open MC form has a higher dipole moment than the closed SP form,^47,48^ however, the results suggest that the Zn complex of MC form has a lower dipole moment than its closed SP counterpart. This would also explain the contrary switching behavior in permittivity after UV and green light treatment.

Based on the UV measurements, it has been shown that exposure to UV light leads to irreversible changes in the absorbance spectra of the samples. To investigate these changes further, IR-spectroscopy was performed on sample 1 : 0.5 before and after subsequent exposure to UV light for 20 min (Fig. 8b). The spectra before and after irradiation do not show any new bands, indicating that no degradation by oxidation occurred. Notably, the band assigned to the C=C stretch showed two absorption maxima for the untreated 1 : 0.5 (1641 and 1666 cm^−1^), but only one for the irradiated sample (1643 cm^−1^). As previously discussed, the band at higher wavenumbers can be assigned to the C=C stretch of the SP, which shifts towards lower wavenumbers when SP is converted to MC. The results indicate that the changes occurring in the sample are not chemical but related to the arrangement of SP in the polymer matrix. This could be due to the aggregation of the MC stacks, which, according to Eckhardt *et al.* can be very stable and irreversible.^46^ The aggregation can lead to shrinking phenomena,^49^ which was also observed in the sample. Another explanation for the observed fatigue is the presence of ZnCl_2_ in the sample. Previous research has demonstrated that ions can stabilize the open MC form, which may impede the molecule's ability to revert to the SP form.^50,51^ These irreversible changes display a significant drawback for the presented system as they would only allow the construction of devices with a functional of a limited amount of cycles and decreasing performance. Further investigations into these stacking phenomena and the means of preventing them would be of interest. Potential methods for this investigation could include X-ray spectroscopy,^52–54^ fluorescence spectroscopy,^55^ or electron microscopy.^46^

## Conclusions

The present study reports on a novel and straightforward synthesis strategy for metallo-supramolecular polymers based on PDMS modified with aminopropyl groups functionalized with spiropyran in a ZnCl_2_-catalyzed reaction. The ZnCl_2_ acts not only as a catalyst but also forms a complex with spiropyran. The spiropyran moieties on the PDMS can form physical cross-links by either spyran-spyran interactions or spiropyran Zn-complex. The metallo-supramolecular polymers behave as thermoplastic elastomers, which can be easily reprocessed. The amount of ZnCl_2_ allows for mechanical properties of the materials to be tuned. Additionally, spiropyran exhibits photo-switching behavior, where exposure to UV light induces ring opening to the merocyanine form, which can be partially reversed by exposure to green light. Notably, the uncomplexed spiropyran shows an increase in the dielectric permittivity upon exposure to UV light, whereas the complexed form shows lower dielectric permittivity values. Although the switching behavior is reversible, the open merocyanine form can stack irreversibly, leading to changes in polymer structure and properties.

## Author contributions

M. B. performed the synthesis, characterization of all materials, and wrote the first draft. T. R. V. conducted the impedance spectroscopy investigations and analysis. D. M. O. initiated the activity, designed the materials, received funding acquisition, and coordinated and supervised this research. All authors contributed with discussions, reviewing, and editing and have approved the final version of the manuscript.

## Conflicts of interest

There are no conflicts to declare.

## Supplementary Material

PY-016-D4PY00964A-s001

## Data Availability

The data, software or code supporting the results reported in a published article can be found in the ESI† and on https://dx.doi.org/10.5281/zenodo.13740447.

## References

[cit1] Shit S. C., Shah P. (2013). Natl. Acad. Sci. Lett..

[cit2] Yilgör E., Yilgör I. (2014). Prog. Polym. Sci..

[cit3] Brook M. A. (2023). Chem. Commun..

[cit4] Barber N., Scarcelli J., Almanza B. A., Daniel J. R., Nelson D. (2007). J. Foodserv..

[cit5] Zare M., Ghomi E. R., Venkatraman P. D., Ramakrishna S. (2021). J. Appl. Polym. Sci..

[cit6] Reynders J. P., Jandrell I. R., Reynders S. M. (1999). IEEE Trans. Dielectr. Electr. Insul..

[cit7] Opris D. M. (2018). Adv. Mater..

[cit8] Sheima Y., Yuts Y., Frauenrath H., Opris D. M. (2021). Macromolecules.

[cit9] Gupta N. S., Lee K. S., Labouriau A. (2021). Polymers.

[cit10] Babu A. R., Gundiah N. (2014). Exp. Mech..

[cit11] Seiffert S., Sprakel J. (2012). Chem. Soc. Rev..

[cit12] Appel E. A., del Barrio J., Loh X. J., Scherman O. A. (2012). Chem. Soc. Rev..

[cit13] Mann J. L., Yu A. C., Agmon G., Appel E. A. (2018). Biomater. Sci..

[cit14] Fujita M. , Molecular Self-Assembly, Organic versus Inorganic Approaches, Springer-Verlag, Berlin, 2000

[cit15] Guo M., Pitet L. M., Wyss H. M., Vos M., Dankers P. Y. W., Meijer E. W. (2014). J. Am. Chem. Soc..

[cit16] Yang B., Zhang H., Peng H., Xu Y., Wu B., Weng W., Li L. (2014). Polym. Chem..

[cit17] Hrabowyj D., Wittenberg L. A., Donahue-Boyle E. M., Chen Y., Brook M. A. (2024). Can. J. Chem..

[cit18] Holten-Andersen N., Harrington M. J., Birkedal H., Lee B. P., Messersmith P. B., Yee K., Lee C., Waite J. H. (2011). Proc. Natl. Acad. Sci. U. S. A..

[cit19] De Greef T. F. A., Smulders M. M. J., Wolffs M., Schenning A. P. H. J., Sijbesma R. P., Meijer E. W. (2009). Chem. Rev..

[cit20] Fawcett A. S., Brook M. A. (2014). Macromolecules.

[cit21] Gallagher N. M., Zhukhovitskiy A. V., Nguyen H. V. T., Johnson J. A. (2018). Macromolecules.

[cit22] Gréger G., Meyer-Zaika W., Böttcher C., Grähn F., Ruthard C., Schmuck C. (2011). J. Am. Chem. Soc..

[cit23] McCoy C. P., Donnelly L., Jones D. S., Gorman S. P. (2007). Tetrahedron Lett..

[cit24] Görner H., Chibisov A. K. (1998). J. Chem. Soc., Faraday Trans..

[cit25] Li M., Zhang Q., Zhou Y. N., Zhu S. (2018). Prog. Polym. Sci..

[cit26] Fagan A., Bartkowski M., Giordani S. (2021). Front. Chem..

[cit27] Natali M., Giordani S. (2012). Chem. Soc. Rev..

[cit28] Xue S. S., Manivannan G., Lessard R. A. (1994). Thin Solid Films.

[cit29] Ramos-Garcia R., Delgado-Macuil R., Iturbe-Castillo D., de Los Santos E. G., Corral F. S. (2003). Opt. Quantum Electron..

[cit30] Garcia J., Addison J. B., Liu S. Z., Lu S., Faulkner A. L., Hodur B. M., Balmond E. I., Or V. W., Yun J. H., Trevino K., Shen B., Shaw J. T., Frank N. L., Louie A. Y. (2019). J. Phys. Chem. B.

[cit31] Kortekaas L., Browne W. R. (2019). Chem. Soc. Rev..

[cit32] Keyvan Rad J., Balzade Z., Mahdavian A. R. (2022). J. Photochem. Photobiol., C.

[cit33] Such G., Evans R. A., Yee L. H., Davis T. P. (2003). J. Macromol. Sci., Polym. Rev..

[cit34] Wübbenhorst M., van Turnhout J. (2002). J. Non-Cryst. Solids.

[cit35] van Turnhout J., Wübbenhorst M. (2002). J. Non-Cryst. Solids.

[cit36] Xue Y., Tian J., Tian W., Zhang K., Xuan J., Zhang X. (2021). Spectrochim. Acta, Part A.

[cit37] Cao W., Wang C., Wang S., Zhang Y., Zhao R. (2021). Chem. Res. Chin. Univ..

[cit38] Fries K. H., Driskell J. D., Samanta S., Locklin J. (2010). Anal. Chem..

[cit39] Racles C., Cozan V., Bele A., Dascalu M. (2016). Des. Monomers Polym..

[cit40] Venkatesan T. R., Gulyakova A. A., Frubing P., Gerhard R. (2018). IEEE Trans. Dielectr. Electr. Insul..

[cit41] Kremer F. and Schönhals A., Broadband Dielectric Spectroscopy, Springer-Verlag, Berlin, 2003

[cit42] Richert R., Yang M. (2003). J. Phys.: Condens. Matter.

[cit43] Venkatesan T. R., Smykalla D., Ploss B., Wübbenhorst M., Gerhard R. (2021). Appl. Phys. A.

[cit44] Tian F., Ohki Y. (2014). J. Phys. D: Appl. Phys..

[cit45] Hammami H., Arous M., Lagache M., Kallel A. (2007). J. Alloys Compd..

[cit46] Eckhardt H., Bose A., Krongauz V. A. (1987). Polymer.

[cit47] Bletz M., Pfeifer-Fukumura U., Kolb U., Baumann W. (2002). J. Phys. Chem. A.

[cit48] Kanj A. B., Chandresh A., Gerwien A., Grosjean S., Bräse S., Wang Y., Dube H., Heinke L. (2020). Chem. Sci..

[cit49] Wismontski-Knittel T., Krongauz V. (1985). Macromolecules.

[cit50] Feuerstein T. J., Müller R., Barner-kowollik C., Roesky P. W. (2019). Inorg. Chem..

[cit51] Ren J., Tian H. (2007). Sensors.

[cit52] Zhang D., Shah P. K., Culver H. R., David S. N., Stansbury J. W., Bowman C. N. (2019). Soft Matter.

[cit53] Fang B., Fan M., Gao H., Wang Y., Gao D., Yu J., Yin M., Dai Y. (2024). J. Mater. Chem..

[cit54] Lydon D. P., Li P., Benniston A. C., Mcfarlane W., Harrington R. W., Clegg W. (2007). Eur. J. Org. Chem..

[cit55] Uznanski P. (2000). Synth. Met..

