# Supplementary material for: Spiropyran-based supramolecular elastomers with tuneable mechanical properties and switchable dielectric permittivity
Source: Polym Chem. 2024 Nov 27;16(2):204–16. doi: 10.1039/d4py00964a (PMC11616703; doi:10.1039/d4py00964a)
Supplement: PY-016-D4PY00964A-s001 [file PY-016-D4PY00964A-s001.pdf]

Supplementary Information (SI) for Polymer Chemistry.
This journal is © The Royal Society of Chemistry 2024

Supplementary information

Spiropyran-based supramolecular elastomers with tuneable mechanical properties and switchable dielectric permittivity

Malte Sebastian Beccard,[a,b] Frank A. Nüesch,[a,c] Thulasinath Raman Venkatesan[a]* and Dorina M. Opris[a,b]*

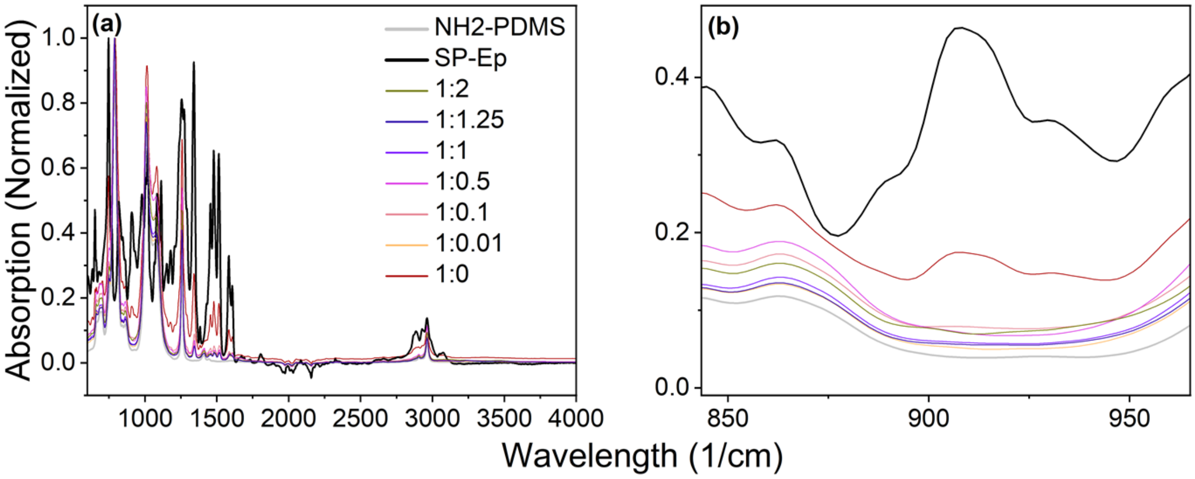

Figure S1 (a) IR spectra of the PDMS-NH_2, SP-Ep, and of the different materials **PDMS-SP-Ep (x:y)**; (b) proof of attachment of SP-Ep to the sample in the presence of a catalyst by the disappearance of the peak at 908 cm^{-1}.

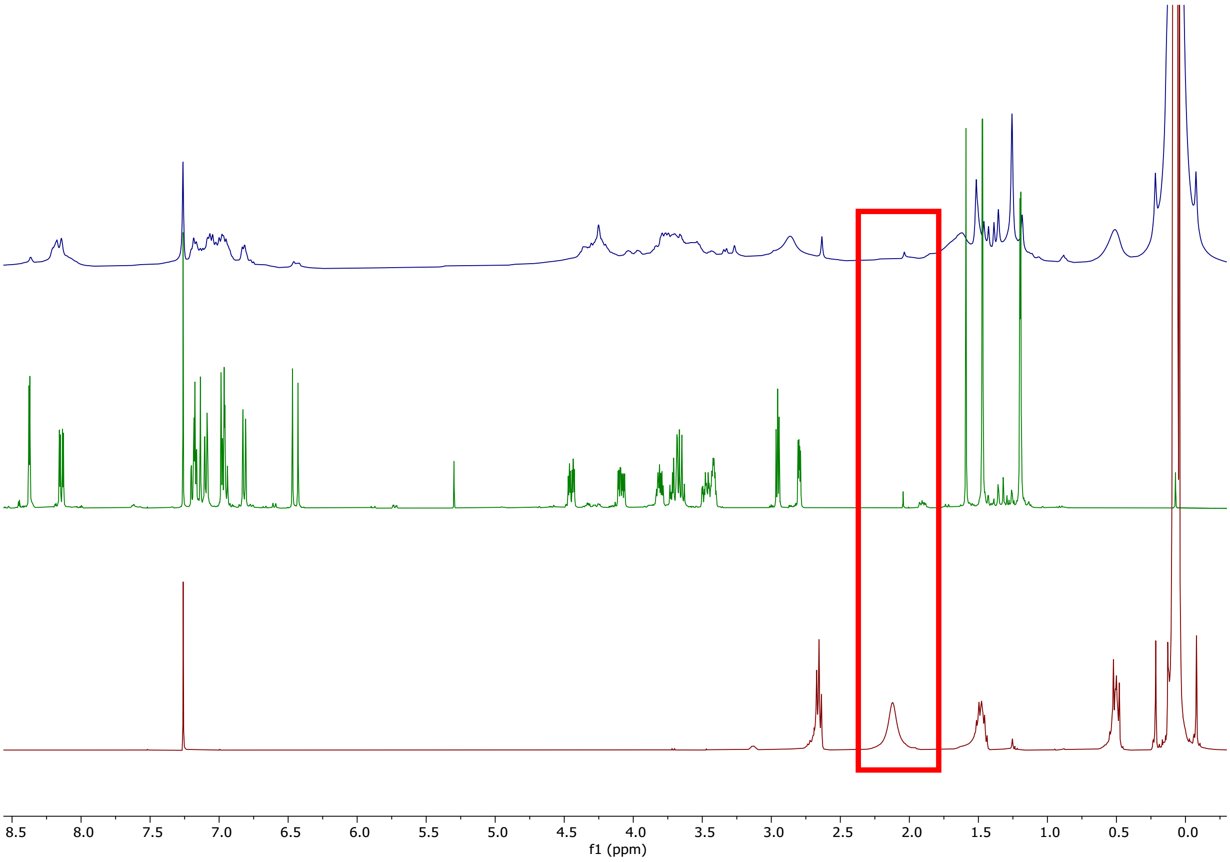

Figure S2 ^{1}H-NMR of SP-EP-(Zn)-PDMS (blue), pure SP-Ep(green), and 5% NH_2-funcionalized (red) PDMS in $CDCl_3$. The red rectangle shows the protonated NH_3^+-group, which disappears after the functionalization of the polymer with SP-Ep. The NMR spectrum of the product looks rather complicated due to the presence of ring-closed spiropyran and opened merocyanine form.

Table S1 Thermogravimetric analysis of different samples and their temperature at a mass loss of 2 %.

	1:1	2:2	0.5:0.5	1:0.1	1:0.01	1:0.5	1:2	1:1.25	1:1.12
Weight loss of 2%	209 °C	196 °C	170 °C	196 °C	236 °C	207 °C	195 °C	196 °C	217 °C

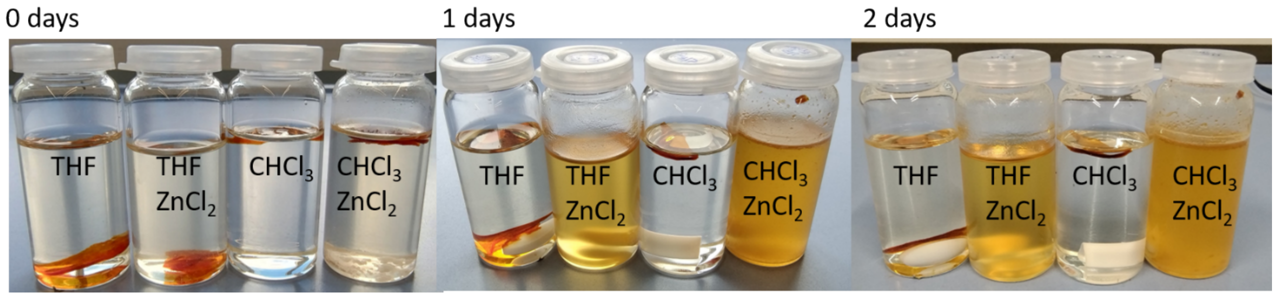

Figure S3 Solubility of sample 1:0.01 in THF and $CHCl_3$ with and without the addition of $ZnCl_2$.

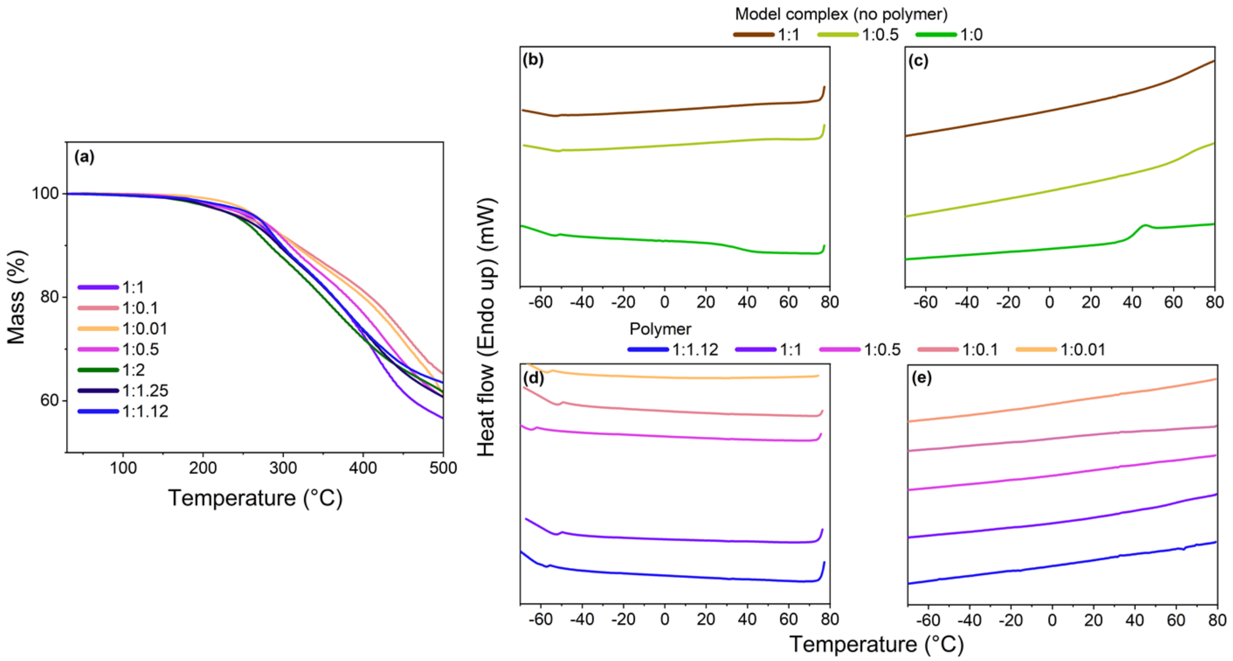

Figure S4 TGA measurements of different samples (a) temperature values at 98 % mass were taken to determine the thermal stability. DSC measurements of pure SP-Ep, and SP-Ep complexes formed with different amounts of $ZnCl_2$ (cooling (b) and 2nd heating (c)), and of different SP-Ep-(Zn)-PDMS samples (cooling (d) and 2nd heating (e)).

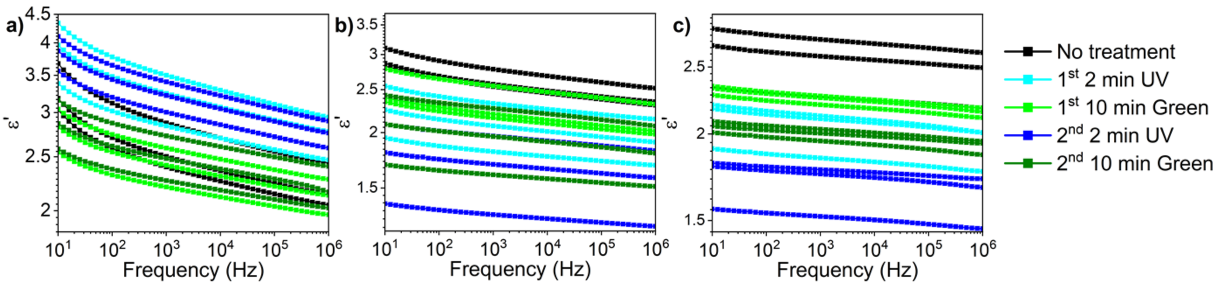

Figure S5 UV-induced switch in the dielectric permittivity: Sample 1:0.01 (a), 1:0.5 (b), and 1:1 before treatment with UV light, after 2 min exposure to UV light and 10 min to green light for two cycles. The absolute values differ from the ones measured without treatment due to handling difficulties and have to be taken as relative values.

Synthesis of 1-(2-hydroxyethyl)-2,3,3-trimethyl-3H-indol-1-ium (1): The synthesis was carried out according to the literature[1] with a few modifications. 2,3,3-Trimethyl-3H-indole (1 eq., 4.92 g, 30.9 mmol), 2-bromoethanol (1.25 eq., 2.7 mL, 38.1 mmol), and acetonitrile (30 mL) were added to a flask equipped with a stirring magnet and a reflux condenser. After refluxing the mixture under an inert atmosphere for 24 h, the solvent was removed, and the product was suspended in n-heptane. The product was subjected to filtration and washing with n-heptane. However, in contrast with the procedure described in the literature, no recrystallization was conducted, as the product was already pure. Following the drying process, the product was obtained in the form of purple crystals, with a yield of 92 % (8.08 g).
^{1}H NMR (400 MHz, d-DMSO, δ): 7.98 (m, 1H); 7.86 (m, 1H); 7.62 (m, 2H); 4.61 (t, 2H); 3.89 (t, 2H); 2.83 (s, 3H); 1.56 (s, 6H) (**Figure S6**).
^{13}C NMR (100 MHz, d-DMSO, δ): 198.24; 142.30; 141.62; 129.78; 129.29; 123.94; 116.07; 58.26; 54.73; 50.76; 22.51 (2C); 14.89 (**Figure S7**).

Synthesis of 9,9,9a-trimethyl-2,3,9,9a-tetrahydrooxazolo[3,2-a]indole (2): The synthesis was carried out according to the literature[1] with some modifications. A solution of KOH (1.6 eq., 2.65 g, 47.2 mmol) in H_2O (140 mL) was prepared and subsequently added to the reagent **1** (1 eq., 8.00 g, 28.5 mmol). After stirring for 20 min at room temperature, the solution was extracted three times with diethyl ether and subsequently dried over $MgSO_4$. The salt was filtered, and the solvent was subsequently evaporated. In contrast with the procedure described in the literature, the product had to undergo purification by column chromatography (gradient of n-heptane to n-heptane:ethylacetate 1:1). The product is in the form of an orange liquid with a yield of 79.9 % (4.63 g).
^{1}H NMR (400 MHz, d-DMSO, δ): 7.08 (m, 2H); 6.82 (m, 2H); 3.73 (m, 2H); 3.48 (m, 1H); 3.34 (m, 1H); 1.33 (s, 3H); 1.28 (s, 3H); 1.09 (s, 3H) (**Figure S8**).
^{13}C NMR (100 MHz, d-DMSO, δ): 151.26; 139.95; 127.71; 122.61; 121.44; 112.17; 108.62; 62.80; 49.61; 46.71; 28.20; 21.03; 17.46 (**Figure S9**).

Synthesis of 2-(3',3'-dimethyl-6-nitrospiro[chromene-2,2'-indolin]-1'-yl)ethan-1-ol (SP-OH): The synthesis was carried out according to the literature[1] with a few modifications. A solution of **2** (1.05 eq., 9.03 g, 44.4 mmol), 2-hydroxy-5-nitrobenzaldehyde (1 eq., 7.05 g, 42.2 mmol) in ethanol (120 mL) was added to a flask equipped with a stirring magnet and a reflux condenser. In contrast with the procedure described in the literature 2-hydroxy-5-nitrobenzaldehyde acts as the limiting reagent, facilitating the subsequent purification process. After refluxing for 3 h under an inert atmosphere, the reaction mixture was allowed to cool to room temperature overnight. The product was filtered and washed with n-heptane. After drying, the product was obtained as purple crystals with a yield of 80.8 % (12.02 g).
^{1}H NMR (400 MHz, d-DMSO, δ): 8.22 (d, 1H); 8.00 (m, 1H); 7.20 (d, 1H); 7.12 (m, 2H); 6.87 (d, 1H); 6.79 (t, 1H); 6.65 (d, 1H); 6.02 (d, 1H); 4.72 (t, 1H); 3.50 (m, 2H); 3.20 (m, 2H); 1.20 (s, 3H); 1.11 (m, 3H) (**Figure S10**).

^{13}C NMR (100 MHz, d-DMSO, δ): 159.65; 147.31; 140.95; 135.94; 128.30; 128.02; 126.14; 123.25; 122.55; 122.11; 119.43; 119.27; 115.89; 106.89 (2C); 59.53; 52.79; 40.09; 26.23; 20.10 (**Figure S11**).

Synthesis of 3',3'-dimethyl-6-nitro-1'-(2-(oxiran-2-ylmethoxy)ethyl)spiro[chromene-2,2'-indoline] (SP-Ep): The synthesis was carried out according to the literature[2]. **SP-OH** (1 eq., 3.55 g, 10.1 mmol), K_2CO_3 (1.05 eq., 1.48 g, 10.7 mmol), and acetonitrile (100 mL) were added to a three-necked flask equipped with reflux condenser and septum. The mixture was set under Ar, using the freeze-pump-thaw technique. After warming to room temperature, 1.5 eq. 2-(bromomethyl)oxirane (1.3 mL, 15.2 mmol) were added under inert conditions. After refluxing for 24 h, the mixture was cooled to room temperature, and the solvent was evaporated. The product was dissolved in ethyl acetate, and the insoluble salts were filtered off. The organic phase was washed three times with water, and the product was purified by column chromatography (ethylacetate:n-heptane 1:2). An orange/brownish solid was obtained with 88.3 % yield (3.64 g).
^{1}H NMR (400 MHz, d-DMSO, δ): 8.42 (d, 1H); 8.19 (m, 1H); 7.28 (d, H); 7.13 (m, 2H); 7.05 (d, 1H); 6.89 (m, 2H); 6.59 (m, 1H); 4.63 (m, 1H); 4.09 (m, 1H); 3.73 (m, 1H); 3.53 (m,1H); 3.39 (m, 2H); 2.89 (t, 1H); 2.77 (q, 1H); 1.40 (s, 3H); 1.11 (s, 3H) (**Figure S12**).
^{13}C NMR (100 MHz, d-DMSO, δ): 161.85; 151.11; 141.64; 139.58; 130.64; 127.93; 126.26; 125.21; 124.98; 123.25; 122.67; 121.71; 113.39; 112.48; 109.80; 70.50; 63.56; 50.07; 49.88; 47.70; 44.09; 28.62; 20.71 (**Figure S13**).

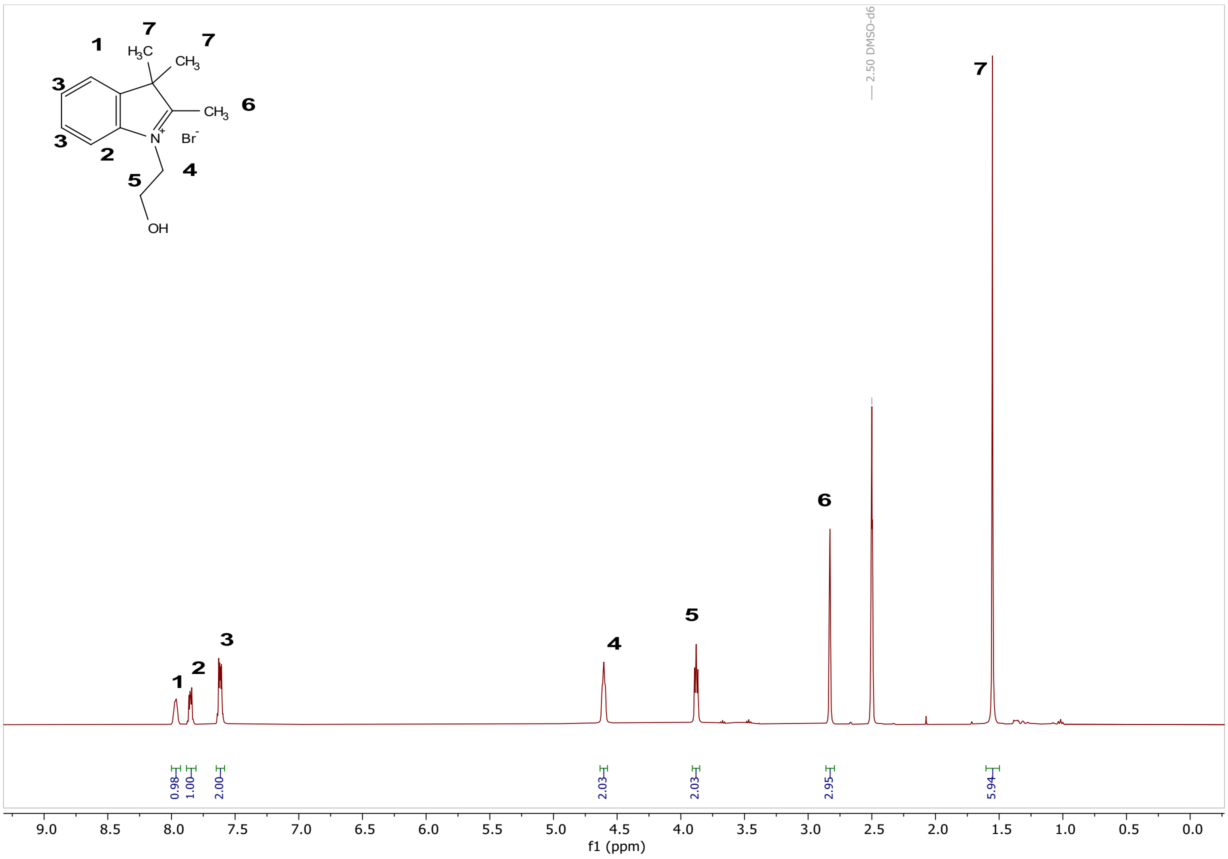

Figure S6 ^{1}H-NMR of 1-(2-hydroxyethyl)-2,3,3-trimethyl-3H-indol-1-ium (**1**) in d-DMSO.

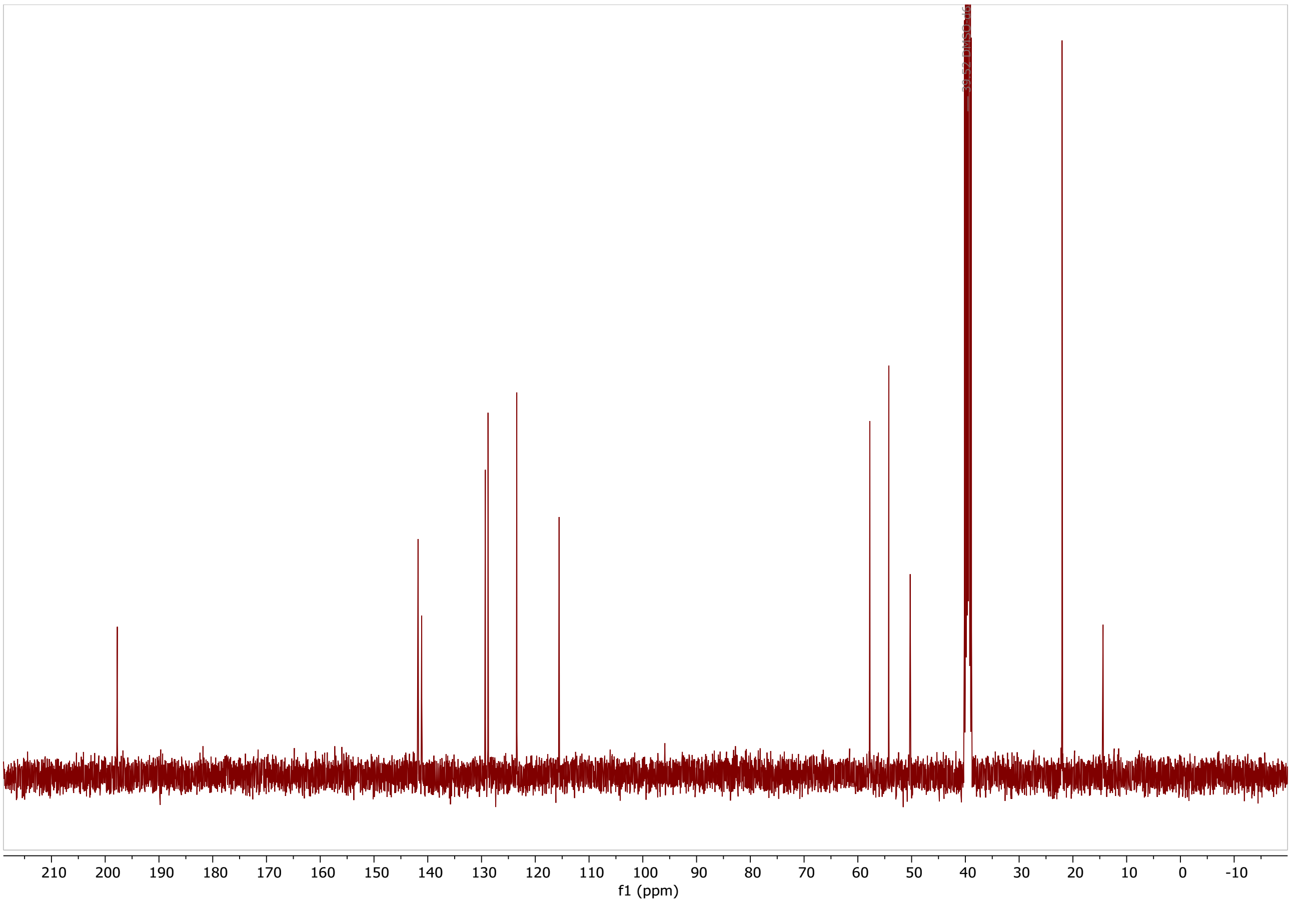

Figure S7 ^{13}C-NMR of 1-(2-hydroxyethyl)-2,3,3-trimethyl-3H-indol-1-ium (**1**) in d-DMSO.

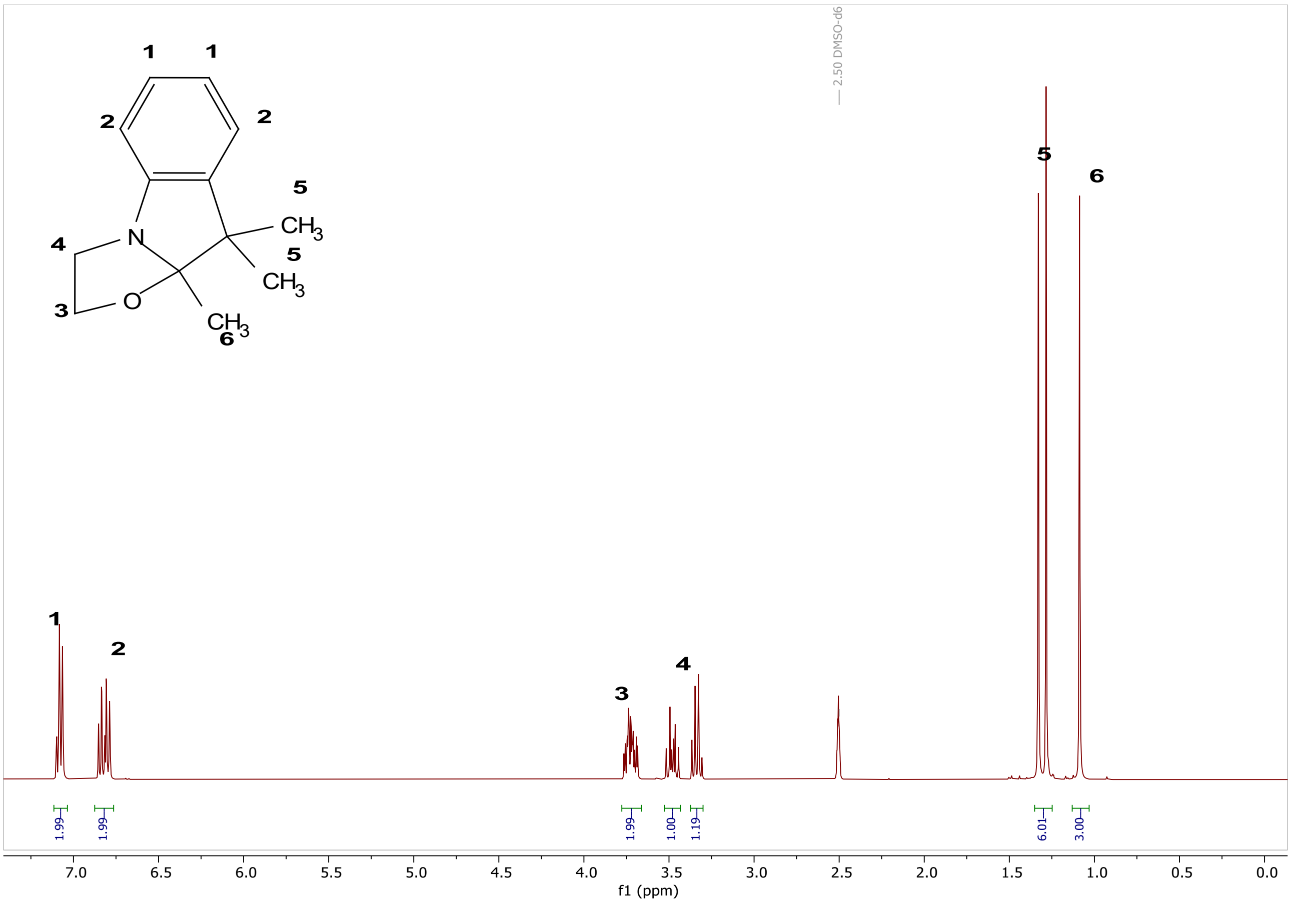

Figure S8 ^{1}H-NMR of 9,9,9a-trimethyl-2,3,9,9a-tetrahydrooxazolo[3,2-a]indole (**2**) in d-DMSO.

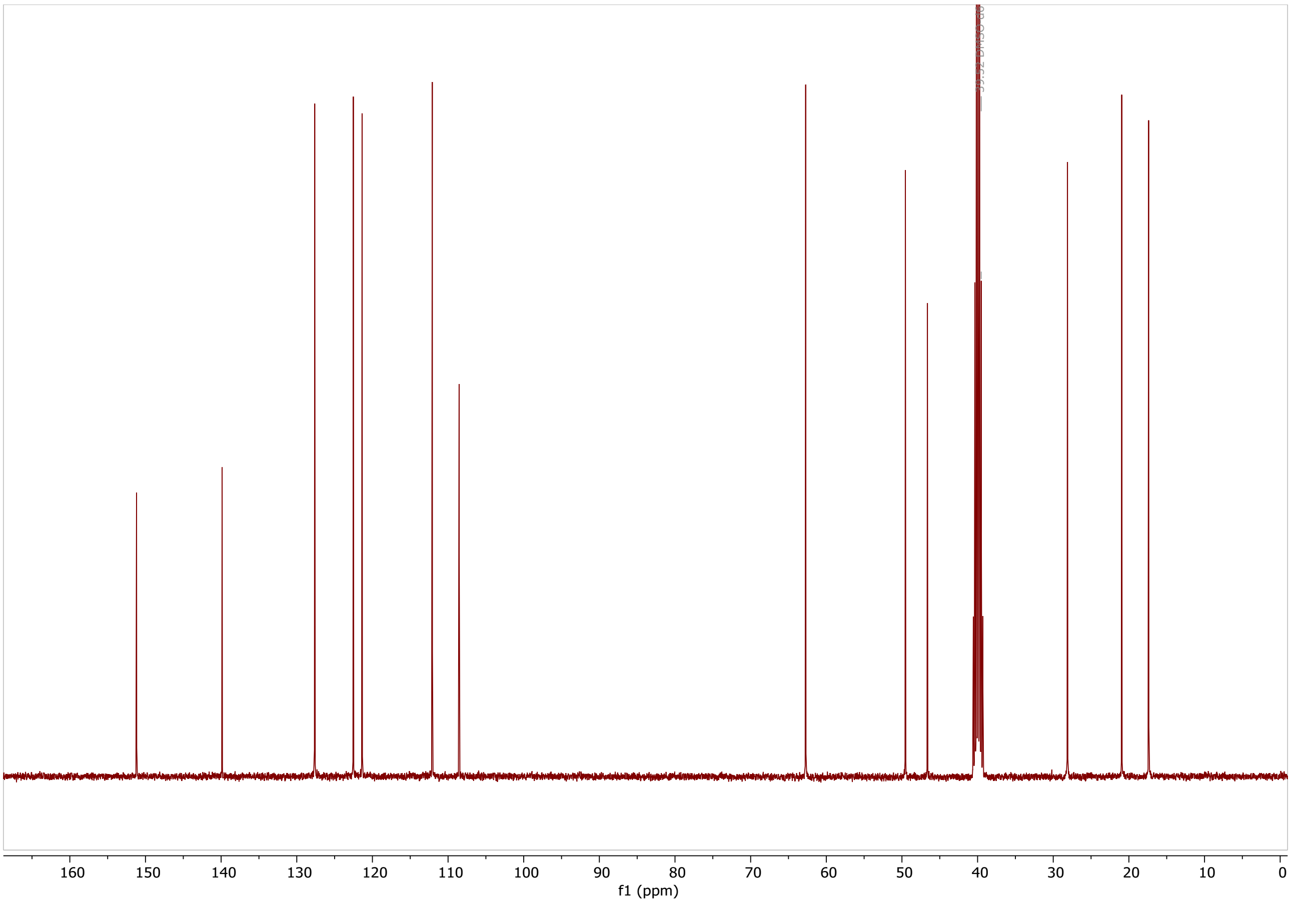

Figure S9 ^{13}C-NMR of 9,9,9a-trimethyl-2,3,9,9a-tetrahydrooxazolo[3,2-a]indole (**2**) in d-DMSO.

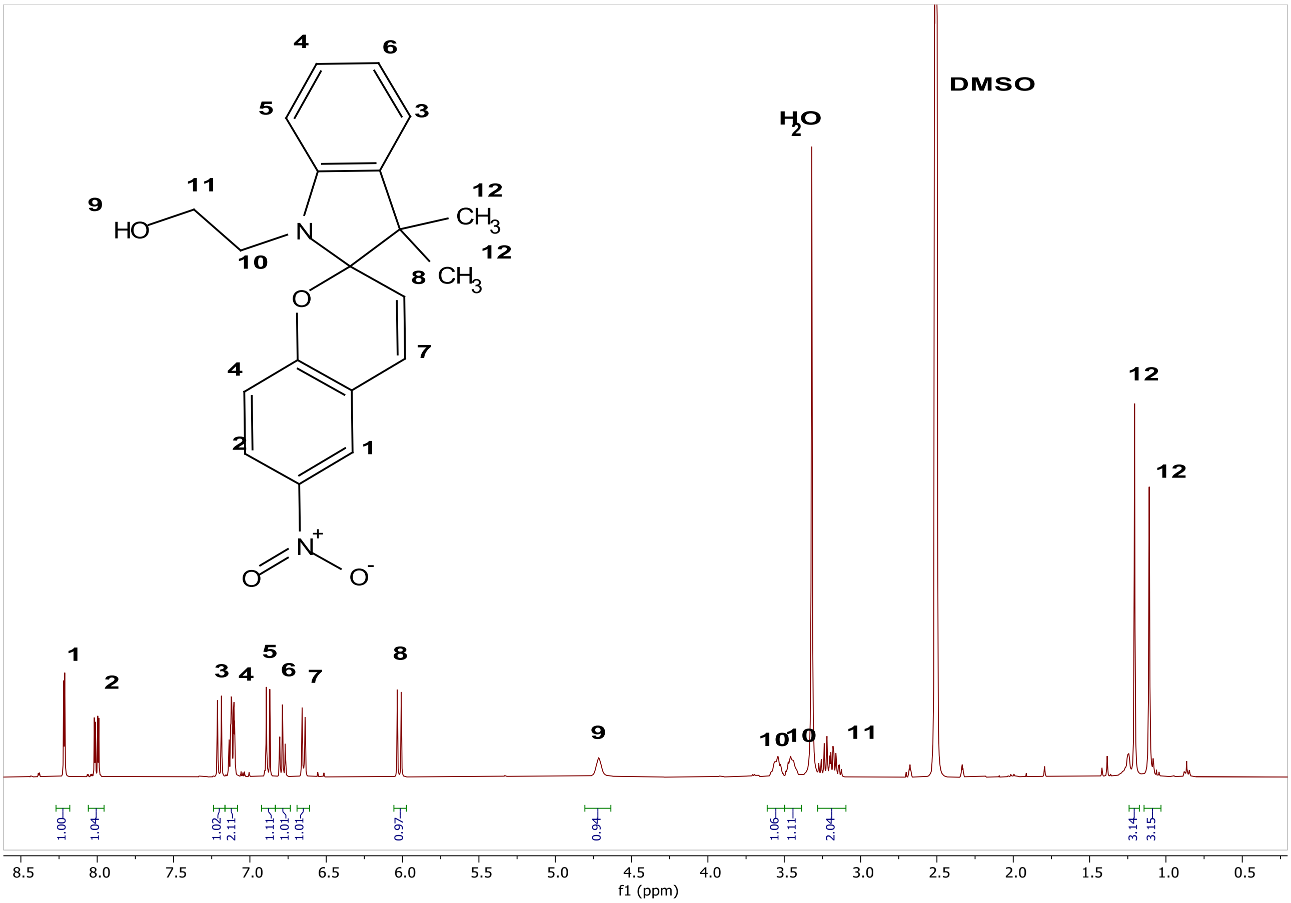

Figure S10 ^{1}H-NMR of 2-(3',3'-dimethyl-6-nitrospiro[chromene-2,2'-indolin]-1'-yl)ethan-1-ol (SP-OH) in d-DMSO.

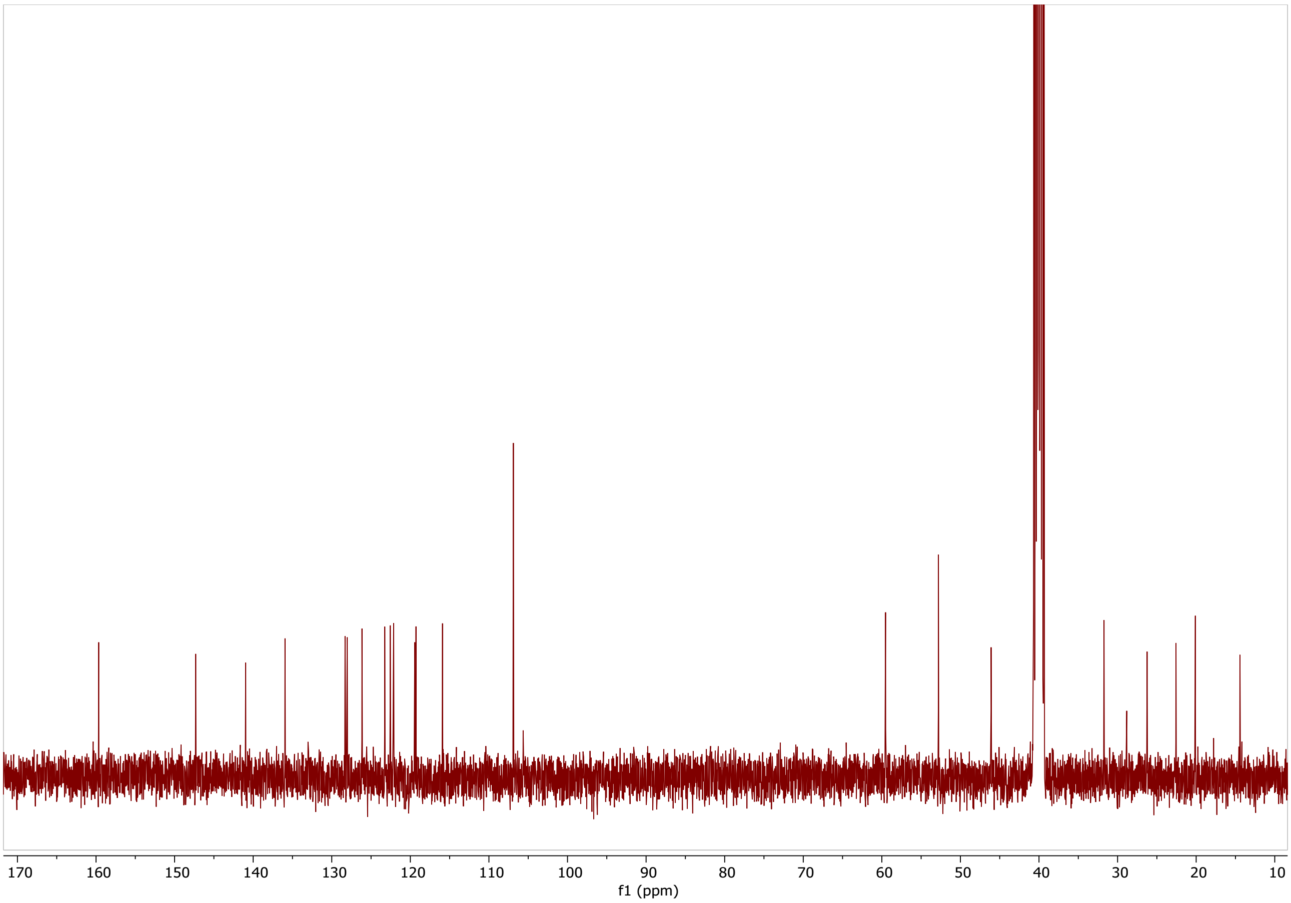

Figure S11 ^{13}C-NMR of 2-(3',3'-dimethyl-6-nitrospiro[chromene-2,2'-indolin]-1'-yl)ethan-1-ol (SP-OH) in d-DMSO.

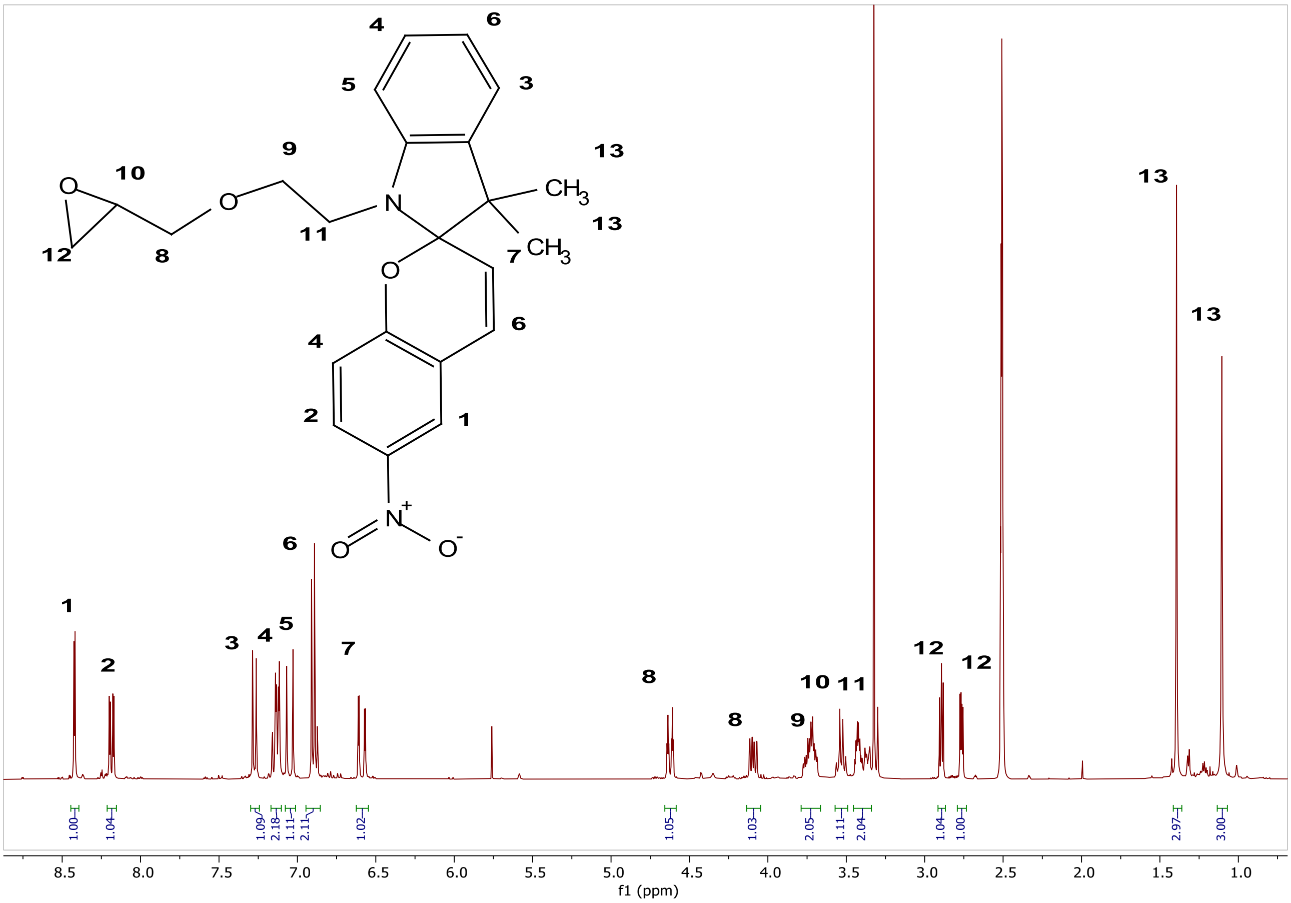

Figure S12 ^{1}H-NMR of 3',3'-dimethyl-6-nitro-1'-(2-(oxiran-2-ylmethoxy)ethyl)spiro[chromene-2,2'-indoline] (SP-Ep) in d-DMSO.

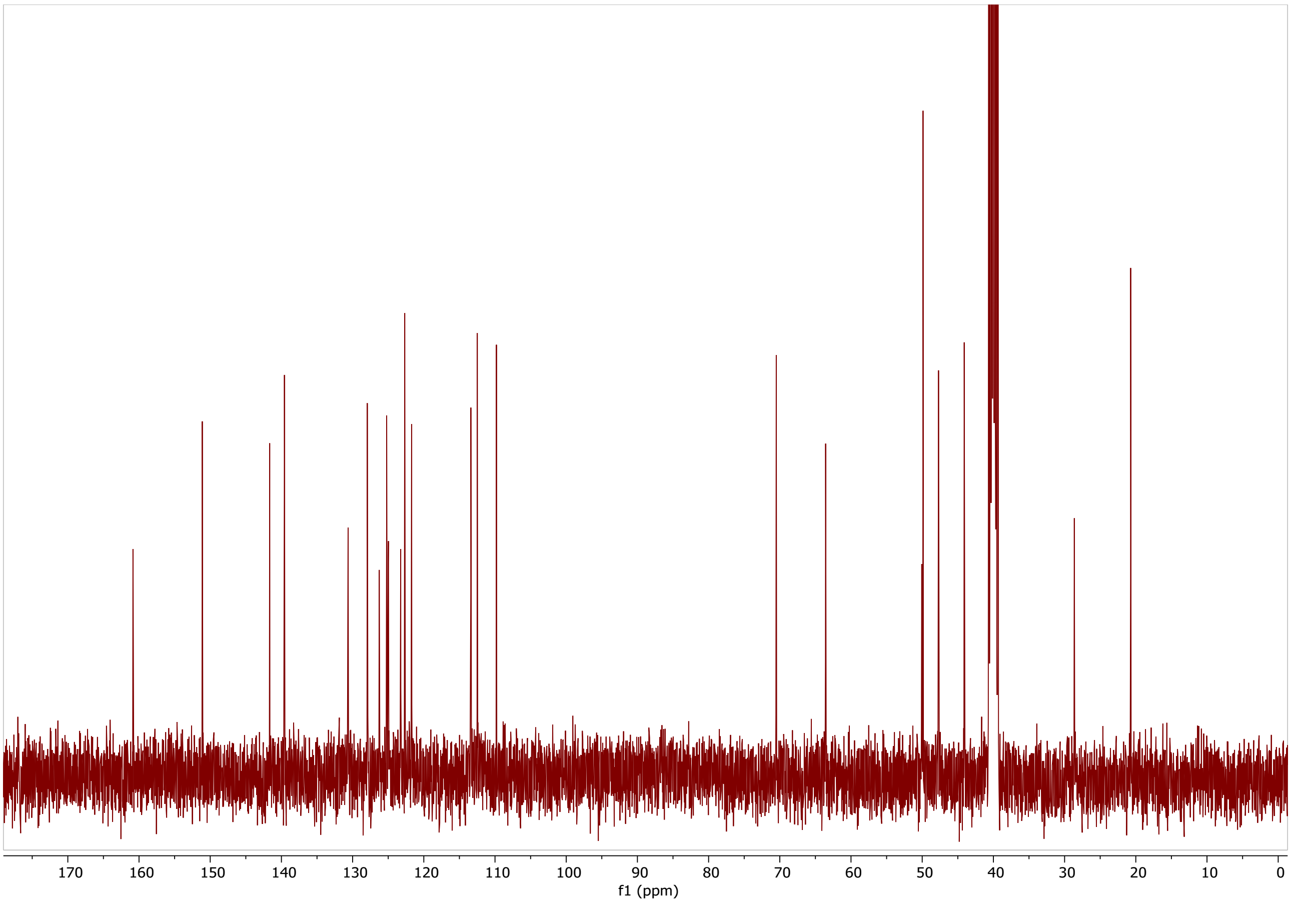

Figure S13 ^{13}C-NMR of 3',3'-dimethyl-6-nitro-1'-(2-(oxiran-2-ylmethoxy)ethyl)spiro[chromene-2,2'-indoline] (SP-Ep) in d-DMSO.

1 Y. Xue, J. Tian, W. Tian, K. Zhang, J. Xuan and X. Zhang, *Spectrochim. Acta - Part A Mol. Biomol. Spectrosc.*, 2021, **250**, 119385.

2 W. Cao, C. Wang, S. Wang, Y. Zhang and R. Zhao, *Chem. Res. Chinese Univ.*, 2021, **37**, 512–521.
